# The Global Nutrient Database: availability of macronutrients and micronutrients in 195 countries from 1980 to 2013

**DOI:** 10.1016/S2542-5196(18)30170-0

**Published:** 2018-08

**Authors:** Josef Schmidhuber, Patrick Sur, Kairsten Fay, Bethany Huntley, Joseph Salama, Alexander Lee, Leslie Cornaby, Masako Horino, Christopher Murray, Ashkan Afshin

**Affiliations:** a Trade and Markets Division, UN's Food and Agricultural Organization, Rome, Italy; b Institute for Health Metrics and Evaluation, University of Washington, Seattle, WA, USA; c Nevada Department of Health and Human Services, Reno, NV, USA

## Abstract

**Background:**

Few data are available on the supply and consumption of nutrients at the country level. To address this data gap, we aimed to create a database that provides information on availability (ie, supply) of 156 nutrients across 195 countries and territories from 1980 to 2013.

**Methods:**

We matched 394 food and agricultural commodities from the Food and Agriculture Organization of the United Nations Supply and Utilization Accounts (SUAs) to food items in the United States Department of Agriculture Food Composition Database and obtained data on nutrient composition of the SUAs' food items. Then, after adjusting for inedible portion of each food item, we added the contributions of individual food items to the availability of each nutrient and estimated the national availability of macronutrients and micronutrients in each year. We validated our estimates by comparing our results with those of national nutrition surveys from three countries (the USA, South Korea, and Ecuador). Using dietary consumption data from the Global Burden of Disease study and two popular machine learning algorithms (Random Forest and XGBoost [extreme gradient boosting]), we developed predictive models to estimate the consumption of each nutrient based on their national availability.

**Findings:**

Globally 2710 kcal (95% UI 2660–2770) were available per person per day in 2013. Carbohydrates were the major contributor to energy availability (70·5%), followed by fats (17·4%), and protein (10·5%). The energy availability and the contribution of macronutrients to total energy widely varied across levels of development. Countries at the higher level of development (high Socio-demographic Index countries) had more energy available per person per day (3270 kcal, 3220–3310); greater contributions from fats (26·0%) and proteins (11·9%) to total energy availability; and lower contributions from carbohydrate (54·8%). During 1980–2013, energy availability and the contributions of protein and fats to energy availability have increased globally and across levels of development while the contribution of carbohydrates to total energy availability has decreased. The supply of the micronutrients has also increased during the same period globally and across levels of development. Our validation analysis showed that, after accounting for waste at the retail and household level, our estimates of macronutrient availability were very close to the consumption data in nationally representative surveys. Our machine-learning models closely predicted the observed intake of nutrients with the out-of-sample correlation of greater than 0·8 between predicted and observed intake for the nutrients included in the analysis.

**Interpretation:**

Our global nutrient database provides a picture of the supply of various nutrients at the country level and can be useful to assess the performance of national food systems in addressing the nutritional needs of their population.

**Funding:**

Bill & Melinda Gates Foundation.

## Introduction

Over the past decade, efforts have been made to estimate the availability of specific nutrients by use of the Food Balance Sheets (FBS) produced by the Food and Agriculture Organization of the United Nations (FAO).^1^, ^2^, ^3^, ^4^ However, no systematic effort has been made to create a comprehensive global database of all macronutrients and micronutrients. Additionally, while useful for providing data on food availability, FBS data are not an ideal source for estimating nutrient availability. The FBS data are compiled by the application of specific conversion factors to data from Supply and Utilization Accounts (SUAs): more detailed lists of food and agricultural items available in each country, which are not in the public domain. Given that the nutrient content of food items (eg, different types of fruits) varies substantially, FBS estimates of nutrient availability might not accurately represent the true national supply of nutrients. Furthermore, previous efforts in this area have not systematically assessed the validity of the estimates by comparing to the actual consumption data.

SUAs provide a comprehensive picture of availability (ie, supply) of food and agricultural commodities across nations and over time.^5^, ^6^, ^7^ These data, if converted into nutrients, can provide a comprehensive picture of the nutrient supply at the national level and will allow the evaluation of how nutrient availability has shifted over time and the identification of drivers of these changes within and across different levels of development. Additionally, such data will allow identification of the food sources of important nutrients in each country and the countries at risk of specific nutrition deficiencies. All this information is important to improve nutrition and achieve the goals of UN Decade of Action on Nutrition.^8^

**Research in context**

**Evidence before this study**

A systematic search of databases was conducted as part of the Global Burden of Disease study 2016. More detailed information about the search strategy has been reported previously (*Lancet* 2017; **390:** 1345–422). Although efforts have been made to estimate the availability of specific nutrients at the country level, there has been no systematic effort to create a comprehensive global database of all macronutrients and micronutrients. Additionally, previous efforts have mostly relied on data from aggregated food groups reported in the Food Balance Sheets of the Food and Agriculture Organization of the United Nations (FAO) and have not systematically assessed the validity of the estimates by comparing them to the actual consumption data.

**Added value of this study**

This study provides a comprehensive picture of nutrient supply at the national level by establishing a global nutrient database and estimating the availability of 156 nutrients in 195 countries and territories from 1980 to 2013. This study, for the first time to our knowledge, uses data from 394 food items reported in the FAO's Supply and Utilization Accounts and we evaluated the validity of our estimates by comparing them with consumption data from nationally representative nutrition surveys from three countries.

**Implications of all the available evidence**

The global nutrient database provides the opportunity to answer important questions about the status of macronutrients and micronutrients across countries, including identification of the countries at risk of specific nutrition deficiencies, identification of the food sources of important micronutrients in each country, and informing national nutrition-sensitive programmes.

We aimed to establish a global database of nutrient availability using FAO SUAs. In this Article, we will provide an overview of the methodological process of creating the database and summarise our key findings at the country level and across different levels of development.

## Methods

### Database construction

We used SUAs from the FAO to estimate the availability (ie, supply) of 156 nutrients in the 195 countries and territories included in the Global Burden of Disease study (GBD) 2016 across 33 years. The list of the nutrients included in our database and the list of the food items included in SUAs are provided in the appendix. The available time series is comprised of estimates from 1961 to 2013. For analytical purposes, and to minimise possible inconsistencies arising from reformulation of various foods, the series was limited to 1980–2013.

SUAs data are compiled annually by the FAO and provide internally consistent information about the supply of up to 394 food and agricultural commodities across nations. For each food and agricultural commodity, SUAs provide an estimate of the available supply to a given population by taking into account production, imports, exports, addition to stocks, use for animal feed and seeds, processing for non-food purposes, and waste or losses at all stages between farms and the household. The methods of data collection and data processing for SUAs have been described in detail previously.^5^, ^6^, ^7^

To obtain data on the nutrient composition of food items in the SUAs, two nutritionists (MH and PS), independently and in duplicate, matched SUAs food items to individual food items catalogued in the United States Department of Agriculture's (USDA) Food and Nutrient Database for Dietary Studies. Given that the FAO SUAs generally represent unprocessed foods available to individuals in a given country and year (eg, chicken meat), the USDA analogues were all raw, uncooked food equivalents (eg, raw whole chicken including meat, skin, giblets, and neck). The proportion of each food item considered to be inedible or simply refuse (the refuse factor) was taken into account and removed from the bulk food item availability. Information on refuse factors was obtained from US Department of Agriculture Handbooks (appendix).^9^, ^10^ Refuse factors were to be considered the same across countries and over time.

After obtaining data on the nutrient composition of each of the 394 food items included in the SUAs and adjusting for inedible portions, we added the contributions of individual food items to the availability of each nutrient to produce an aggregate measure of nutrient availability within each country and for each year of available data. The per capita availability of every nutrient *i* in year *t* and country *c* can be expressed as:

$$NUT_{itc}\;=\;\sum_{j=1}^{394}NUT_{jitc}$$

where *j* is the SUA food item, *t* ranges from 1980 to 2013, and *c* ranges from 158 to 178 (depending on year). SUAs data were not available for 18 countries and territories: American Samoa, Andorra, Bahrain, Bhutan, Equatorial Guinea, Eritrea, the Federated States of Micronesia, Greenland, Guam, the Marshall Islands, the Northern Mariana Islands, Palestine, Puerto Rico, Qatar, Singapore, South Sudan, Tonga, and the Virgin Islands (US).

We used spatiotemporal Gaussian process regression to produce a full time-series of estimates for all nutrients across all 195 countries and territories. Spatiotemporal Gaussian process regression is a powerful method for estimating non-linear trends and allows the borrowing of strength across geography and time. This modelling approach has been described in detail previously^11^ and we have summarised the process in the appendix. To improve our estimates in data-sparse countries, we used the 10 year lag-distributed national income per capita as a covariate in our model.

Energy availability in a given country and year was presented in the form of kcal per person per day. In alignment with the USDA's Food and Nutrient Database for Dietary Studies, we used the Atwater approach^10^ to estimate the energy availability. This approach involves assigning 4 kcal for every 1 g of protein, 4 kcal for every 1 g of carbohydrate, 9 kcal for 1 g of fat, and 7 kcal for every 1 g of alcohol.^12^ The energy estimated through this approach represents the energy available after losses from digestive and urinary processes.

Our calculation of the total digestible carbohydrates available in each food item was based on the difference in total energy of the food item and the energy content of the food item from protein, fats, and alcohol, as worked out with the Atwater approach. This approach allowed us to exclude indigestible dietary fibres that do not contribute to energy. Thus, our carbohydrate estimates represent all monosaccharides (galactose, glucose, and fructose), disaccharides (sucrose, lactose, and maltose), and starches.

For each nutrient, we estimated the mean value and the corresponding 95% uncertainty interval (UI), which was estimated based on the observed variance in the data between countries over time.

### Relationship between availability and consumption of nutrients

To validate our estimates of macronutrient availability, we compared them with macronutrient consumption data estimated from 24 h dietary recall surveys in eight cycles (1999–2014) of the United States National Health and Nutrition Examination Survey (NHANES),^13^, ^14^, ^15^, ^16^, ^17^, ^18^, ^19^, ^20^ ten cycles (1998–2013) of the Korea National Health and Nutrition Examination Survey (KNHANES),^21^, ^22^, ^23^, ^24^, ^25^, ^26^, ^27^, ^28^, ^29^, ^30^ and one cycle (2012) of the Ecuador National Health and Nutrition Survey.^31^ To account for waste at the retail and household level and make availability and consumption data more comparable, we estimated the percentage of energy from each macronutrient in both our database and dietary recall surveys.

To evaluate the possibility of using our nutrient availability database for predicting nutrient consumption, we developed three separate prediction models to estimate the intake of selected nutrients (calcium, fibre, polyunsaturated fat, saturated fat, zinc) based on their estimates of availability in our database. In this analysis, we first characterised the age and sex pattern of intake for each nutrient using the nationally representative 24 h dietary recall data from the GBD Diet Database and the GBD risk factor modelling framework.^11^ We then applied the age and sex patterns of intake for each nutrient to the nutrient availability estimates and estimated the nutrient availability by age and sex for each country-year. Then, we matched nutrient availability data to the nutrient intake data by age, sex, country, and year and did three separate analyses to estimate consumption based on the nutrient availability. We first did a linear mixed-effects regression analysis to estimate the nutrient intake based on nutrient availability by age (*a)*, sex (*s*), country (*c*), and year (*t*) using the following equation:

$$Nutrient\;Intake_{c,a,s,t}\;=\;\beta \;*\;Nutrient\;Availability_{c,a,s,t}\;+\;age\;+\;sex\;+\;\alpha_{\sup er-region}$$

where super-region represents the super-regions included the GBD.^11^

To optimise our predictive models, we used the same dataset to train two machine learning models that included the same parameters. The first machine learning approach that we used was Random Forest,^32^, ^33^ which is a popular learning technique that uses an ensemble of fully developed decision trees on bootstrapped samples. The second approach that we used was XGBoost (extreme gradient boosting).^34^ XGBoost is a learning technique that uses decision trees to produce an ensemble of weak prediction models. We used out-of-sample root mean-square error and out-of-sample correlation to assess the performance of our models and select the best performing model.

### Relationship between availability of macronutrients and development

To evaluate relationships between the supply of various macronutrients, we assessed pairwise Pearson correlations for all macronutrients across 195 countries and territories. To characterise the potential patterns of macronutrient replacement, we estimated the Pearson correlation between the percentage of energy available from each pair of macronutrients. We used Socio-demographic Index (SDI) to assess the level of development for each country. SDI is a composite indicator that was developed as part of the GBD study, and uses lag-distributed income per person, average educational attainment in the population older than 15 years, and the total fertility rate to provide a measure of development for a given country. Each component of the SDI was first rescaled to a value between 0 and 1, with 0 being the lowest (worst) value observed in the time period of the study and 1 being the highest (best) value observed. SDI was then computed as the geometric mean of these three rescaled components. To position countries across the development continuum, we used SDI quintiles and categorised countries to five levels of development: low, lower-middle, middle, upper-middle, and high.

### Role of the funding source

The funder of the study had no role in study design, data collection, data analysis, data interpretation, or writing of the report. The first author and the corresponding author had full access to all the data in the study and had final responsibility for the decision to submit for publication.

## Results

An interactive data visualisation of availability of all nutrients in 195 countries and territories between 1980 and 2013 is publicly available online. Here we highlight the findings related to macronutrients and selected micronutrients (iron, vitamin A, and zinc) at the global level, across levels of development, and among the 15 most populous countries in 2013 (China, India, USA, Indonesia, Brazil, Pakistan, Nigeria, Bangladesh, Russia, Japan, Mexico, Philippines, Ethiopia, Vietnam, and Egypt, comprising two-thirds of the world's population.

**Figure 1:**
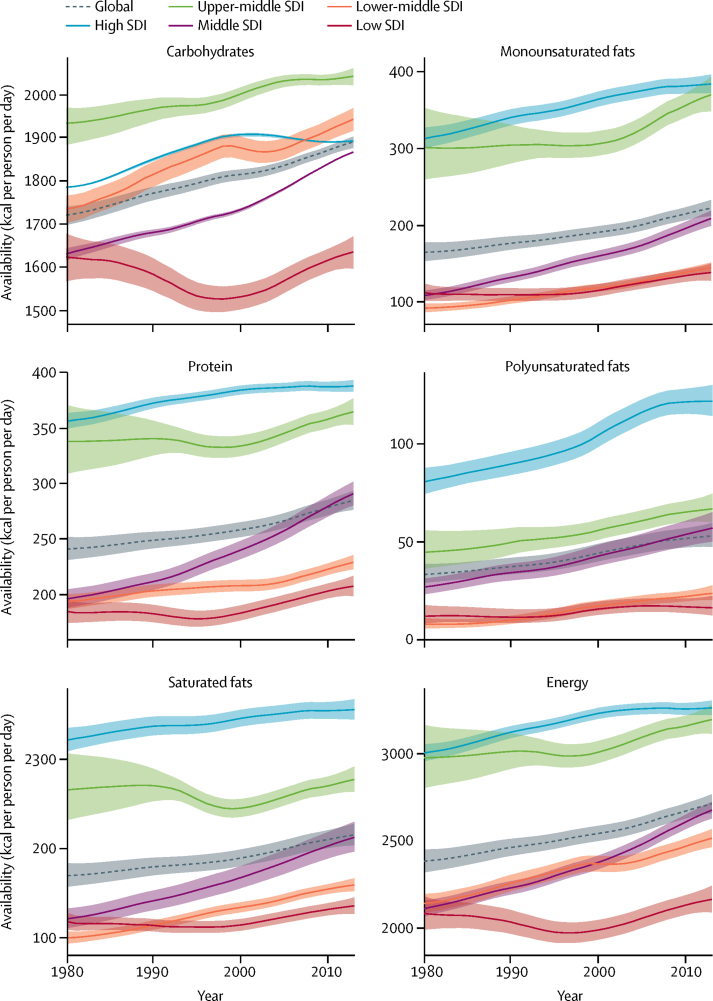
Availability of energy and macronutrients at globally and across levels of development, 1980–2013

**Figure 2:**
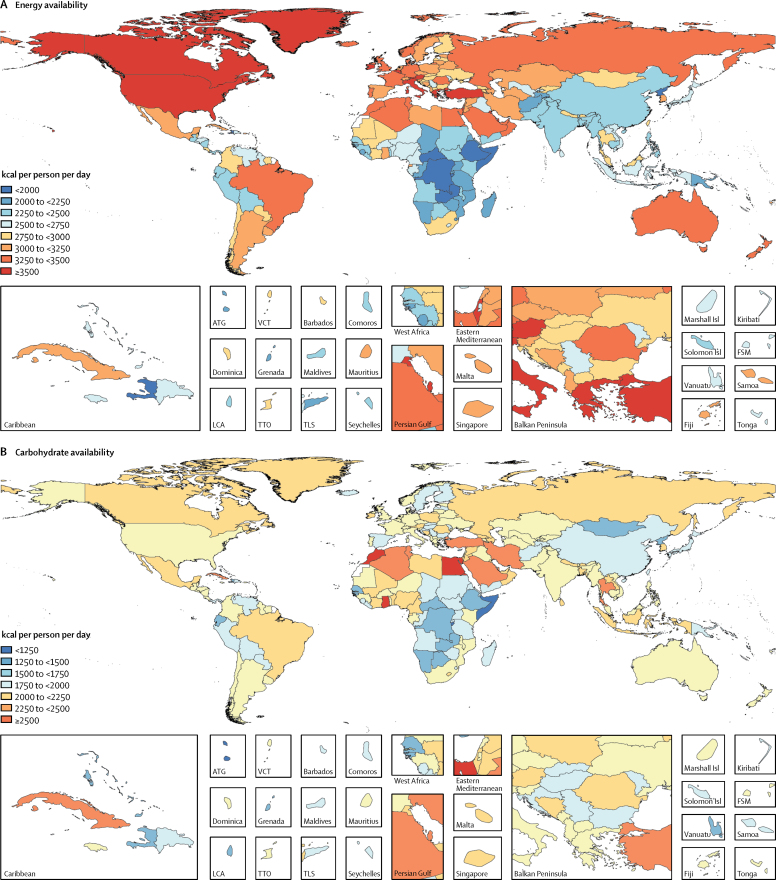

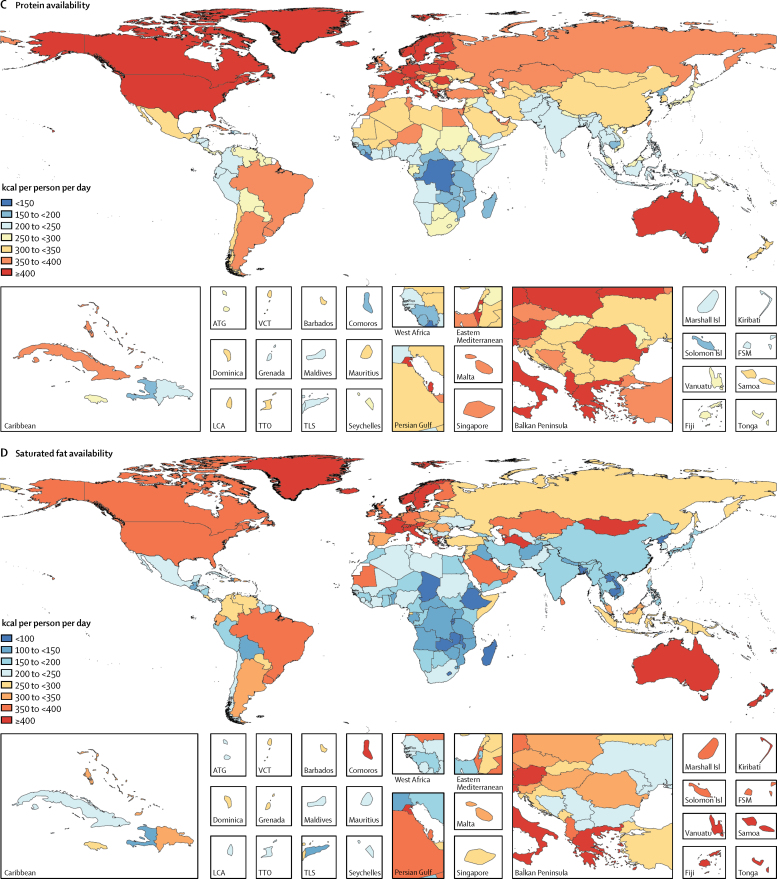

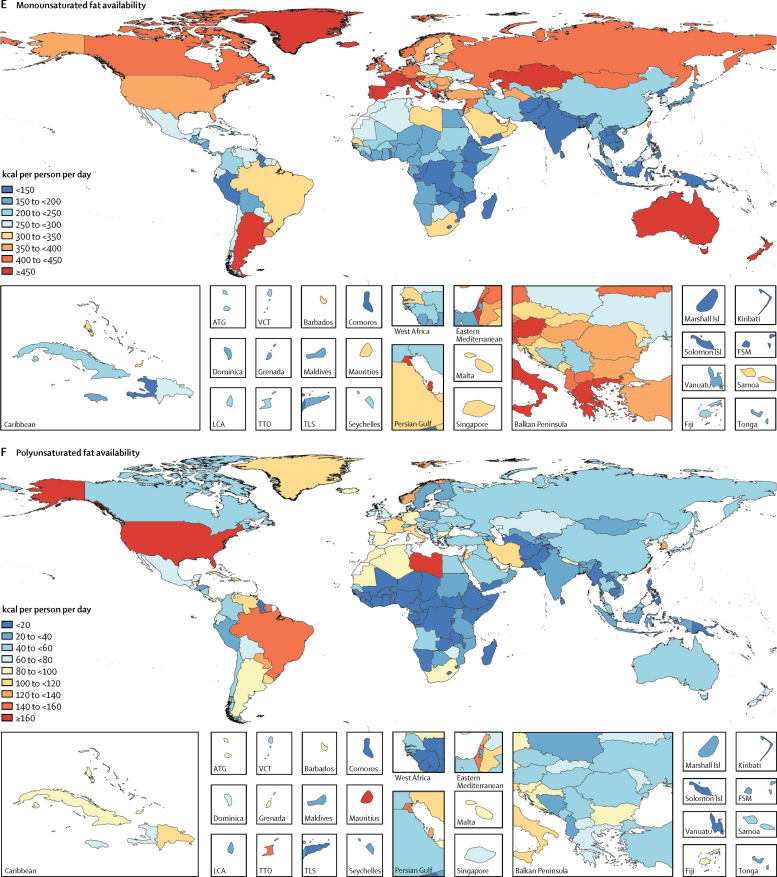
Availability of energy and macronutrients by country in 2013 Maps show availability of (A) energy, (B) carbohydrate, (C) protein, (D) saturated fats, (E) monounsaturated fats, and (F) polyunsaturated fats. ATG=Antigua and Barbuda. VCT=Saint Vincent and the Grenadines. LCA=Saint Lucia. TTO=Trinidad and Tobago. Isl=Islands. FSM=Federated States of Micronesia. TLS=Timor-Leste.

Globally 2710 kcal (95% UI 2660–2770) were available per person per day in 2013 (figure 1). The energy availability widely varied across levels of development ranging from 2170 kcal (2090–2250) per person per day in low-SDI countries to 3270 (3220–3310) kcal per person per day in high-SDI countries. Among the most populous countries (figure 2), the highest level of energy availability was in the USA (3500 kcal [3450–3560] per person per day) and the lowest was in Ethiopia (1880 kcal [1810–1940] per person per day).

During 1980–2013, global energy availability increased from 2390 kcal per person per day to 2710 kcal per person per day; a net increase of 320 kcal per person per day (figure 1). Across levels of development, the highest absolute increase in energy availability was in the middle-SDI (570 kcal per person per day) and lower-middle-SDI countries (380 kcal per person per day), whereas energy availability in low-SDI countries has increased by only 80 kcal per person per day. Among the most populous countries, the largest absolute increases in energy availability were in Nigeria (880 kcal per person per day) and Brazil (710 kcal per person per day), while Japan showed the smallest increase in energy availability (40 kcal per person per day).

In 2013, 1890 kcal (95% UI 1880–1900) from carbohydrates were available per person per day (figure 1). The highest availability of carbohydrates was in upper-middle-SDI countries (2040 kcal [2020–2060] per person per day) and the lowest was in low-SDI countries (1640 kcal [1600–1670] per person per day). Across the most populous countries, the highest availability of carbohydrates was observed in Egypt (2730 kcal [2690–2770] per person per day) and the lowest was in Ethiopia (1430 kcal [1390–1470] per person per day).

Between 1980 and 2013, the global availability of carbohydrates increased by 170 kcal per person per day. Across levels of development, the highest increase in carbohydrate availability was in middle-SDI countries (230 kcal per person per day) and the smallest increase was in low-SDI countries (10 kcal per person per day; figure 1). Among the most populous countries, the largest increases in carbohydrate availability were in Nigeria (720 kcal per person per day) and Egypt (380 kcal per person per day), while carbohydrate availability decreased in Pakistan (−20 kcal per person per day), Mexico (−55 kcal per person per day), and Japan (−85 kcal per person per day) in this period.

Globally, 285 kcal (95% UI 275–295) per person per day were available from protein in 2013 (figure 1). In high-SDI countries, protein availability was 390 kcal (385–395) per person per day, whereas in low-SDI countries it was 210 kcal (200–220) per person per day. Among the most populous countries, the highest availability of protein was in the USA (420 kcal [415–425] per person per day) and the lowest in Bangladesh (200 kcal [195–205] per person per day; figure 2).

In 1980–2013, the availability of protein increased by 45 kcal per person per day globally (figure 1). The largest increase in protein availability was in middle-SDI countries (95 kcal per person per day), whereas low-SDI countries showed little increase in protein availability. Having started in 1980 at 185 kcal per person per day, protein in low-SDI countries increased by only 25 kcal per person per day. Protein availability has increased among all the most populous countries. The largest increases in protein availability were in Brazil (125 kcal per person per day) and China (115 kcal per person per day) and the lowest increases were in Mexico and India (both 25 kcal per person per day).

Globally in 2013, 225 kcal (215–235) per person per day were available from monounsaturated fats, 55 kcal (50–60) per person per day from polyunsaturated fats, and 215 kcal (200–230) per person per day from saturated fats (figure 1). Across levels of development, the highest availabilities of monounsaturated fats (385 kcal [370–395] per person per day), polyunsaturated fats (120 kcal [115–130] per person per day), and saturated fats (355 kcal [345–365] per person per day) were in high-SDI countries and the lowest availabilities were in low-SDI countries (140 kcal [130–150] per person per day from monounsaturated fats, 15 kcal [10–20] per person per day from polyunsaturated fats, and 135 kcal [125–145] per person per day from saturated fats). Among the most populous countries, the highest availability of monounsaturated fat (400 kcal [390–405] per person per day), polyunsaturated fat (200 kcal [195–210] per person per day), and saturated fats (385 kcal [380–390] per person per day) were in the USA. The lowest availabilities of monounsaturated fat (80 kcal [75–85] per person per day) and saturated fat (90 kcal [85–95] per person per day) were in Bangladesh and the lowest availability of polyunsaturated fats was in Nigeria (<10 kcal per person per day).

During 1980–2013, among different types of fats, the largest global increase was for monounsaturated fats (55 kcal per person per day), followed by saturated fats (45 kcal per person per day), and polyunsaturated fats (20 kcal per person per day). Across levels of development, the largest increases in the availability of monounsaturated fats (100 kcal per person per day) and saturated fats (95 kcal per person per day) were in middle-SDI countries and the largest increase in the availability of polyunsaturated fats (40 kcal per person per day) was in high-SDI countries. Among the most populous countries, the largest increases in the availability of monounsaturated fats (155 kcal per person per day) and saturated fats (180 kcal per person per day) were in Brazil, whereas the largest increase in the availability of polyunsaturated fat (55 kcal per person per day) was in the USA.

**Figure 3:**
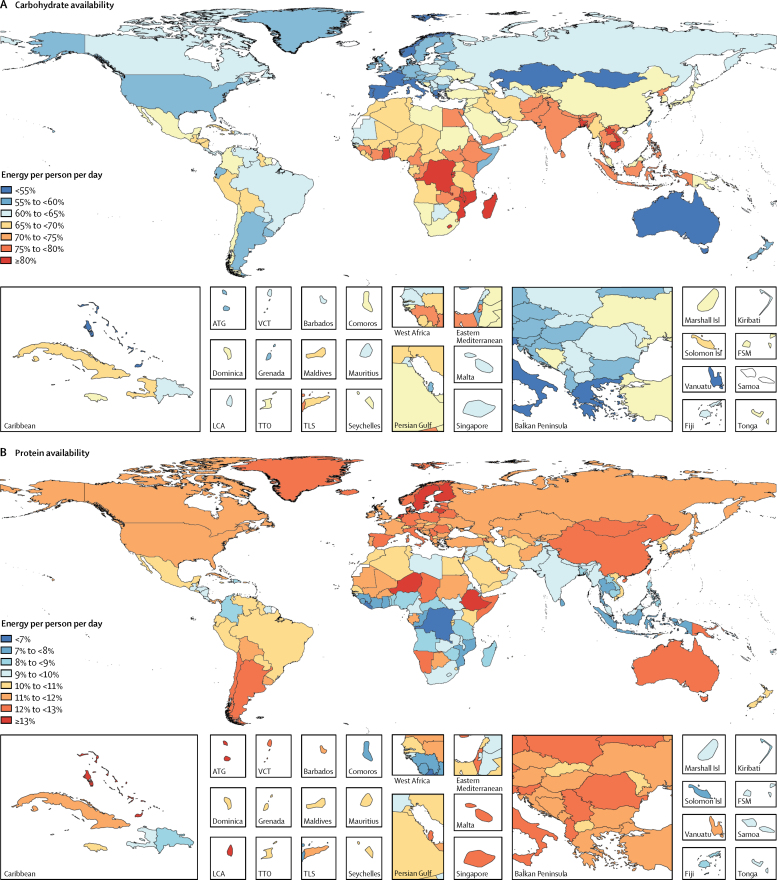

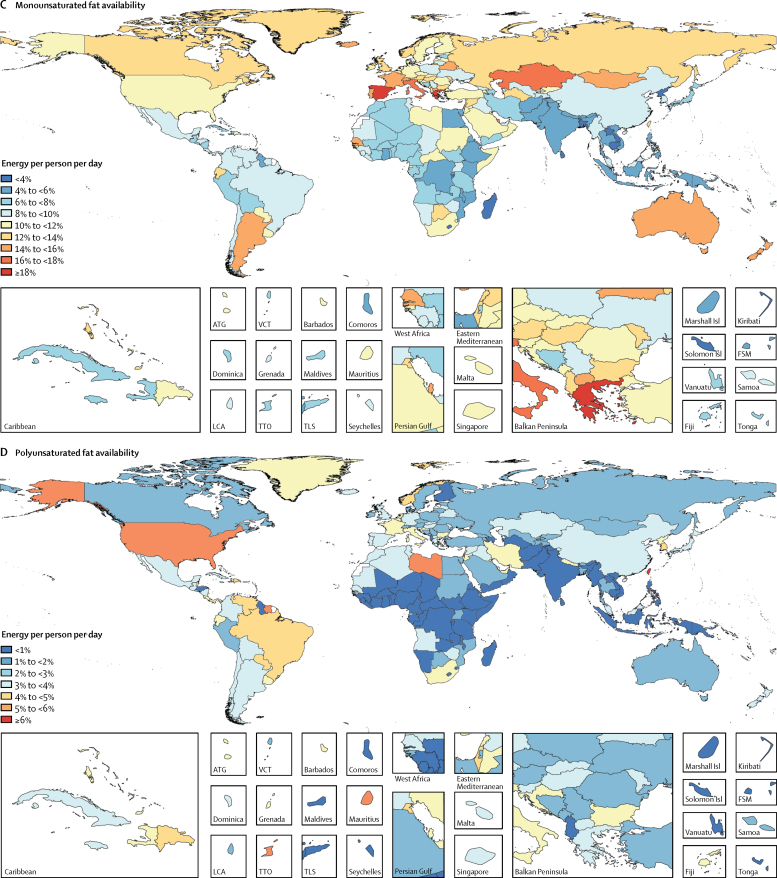

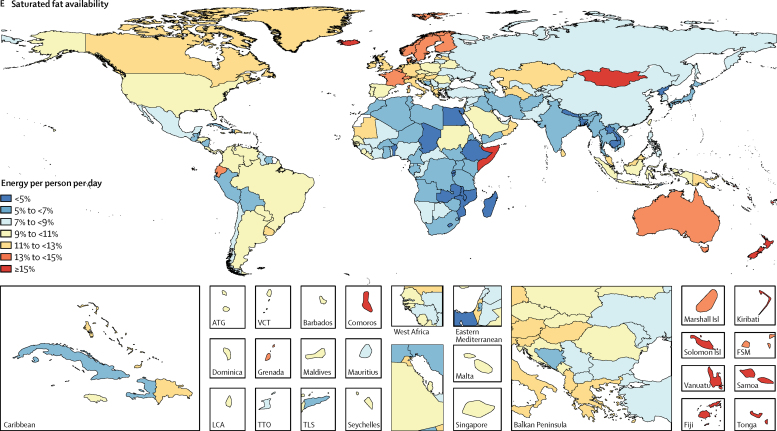
Contribution of macronutrients to energy availability by country in 2013 Maps show contribution of (A) carbohydrate, (B) protein, (C) monounsaturated fats, (D) polyunsaturated fats, and (E) saturated fats to energy availability. ATG=Antigua and Barbuda. VCT=Saint Vincent and the Grenadines. LCA=Saint Lucia. TTO=Trinidad and Tobago. Isl=Islands. FSM=Federated States of Micronesia. TLS=Timor-Leste.

Globally 70·5% (95% UI 69·5–71·4) of total energy available per person per day was from carbohydrates, followed by fats (17·4%, including 7·9% [7·7–8·1] monounsaturated fats, 1·8% [1·7–2·0] polyunsaturated fats, and 7·7% [7·4–8·0] saturated fats) and protein (10·4% [10·3–10·5]). Countries at the higher SDI level had lower contributions from carbohydrate and greater shares of fats and proteins. The highest shares of monounsaturated fats (11·6% [11·4–11·8] of total energy availability), polyunsaturated fats (3·7% [3·5–3·9]), saturated fats (10·7% [10·5–10·9]), and protein (11·89% [11·88–11·90]) were in high-SDI countries, whereas the highest contributions from carbohydrate were in lower-middle-SDI countries (77·2% [76·6–77·7]) and low-SDI countries (75·7% [74·7–76·6]). Among the most populous countries, Bangladesh (83·8%) and the Democratic Republic of the Congo (80·7%) had the highest shares of carbohydrates; Ethiopia (13·5%) and China (12·2%) had the highest shares of protein; Russia (12·2%) and Germany (11·9%) had the highest shares of monounsaturated fat; the USA (5·7%) and Brazil (4·5%) had the highest shares of polyunsaturated fat; and Germany (11·7%) and the USA (11%) had the highest shares of saturated fats (figure 3). The smallest shares were in Germany for carbohydrate (54·9%), the Democratic Republic of the Congo for protein (4·4%), Bangladesh for monounsaturated fats (3·3%), Pakistan for polyunsaturated fats (<1%), and Bangladesh for saturated fats (3·6%).

**Figure 4:**
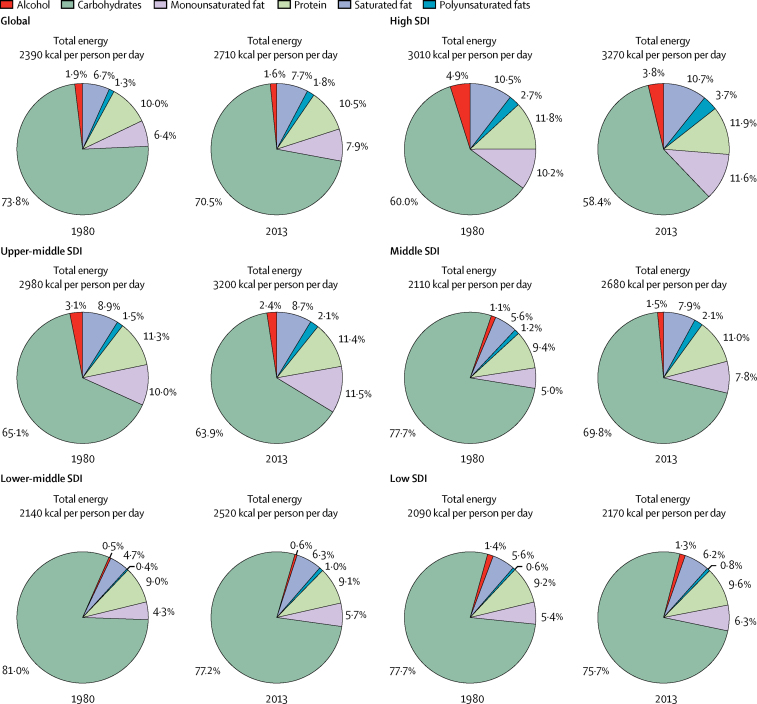
Contribution of macronutrients to energy availability globally and across levels of development, 1980–2013 SDI=Socio-demographic Index.

Since 1980, the contribution of carbohydrates to total energy availability has decreased globally and across all levels of development, while the contributions of protein and fats have increased (figure 4). The largest decrease in the proportion of energy from carbohydrates and the largest increase in the proportion of energy from fats and protein were in middle-SDI countries (figure 4). High-SDI countries have shown an increase in the availability of polyunsaturated fat and monounsaturated fat while maintaining that of saturated fat at a relatively stable level. However, other countries have had an increase in availability of all forms of fats. Among the most populous countries, the contribution of carbohydrates to total energy availability has decreased in all countries, except for Nigeria. The largest decreases in the contributions from carbohydrates were in Vietnam, China, and Brazil. The contributions from protein increased in all countries other than the USA, Russia, and India. The largest increases in the contributions from protein were in China, Vietnam, and Brazil. The contributions from monounsaturated fats, polyunsaturated fats, and saturated fats increased in the majority of the most populous countries, with the exception of Nigeria for monounsaturated fat; Nigeria and Ethiopia for polyunsaturated fat; and Russia, Nigeria, Egypt, and the USA for saturated fats. The largest increases in the contributions from monounsaturated fats and polyunsaturated fats were in China, whereas Brazil had the largest increase in the contributions from saturated fats.

**Figure 5:**
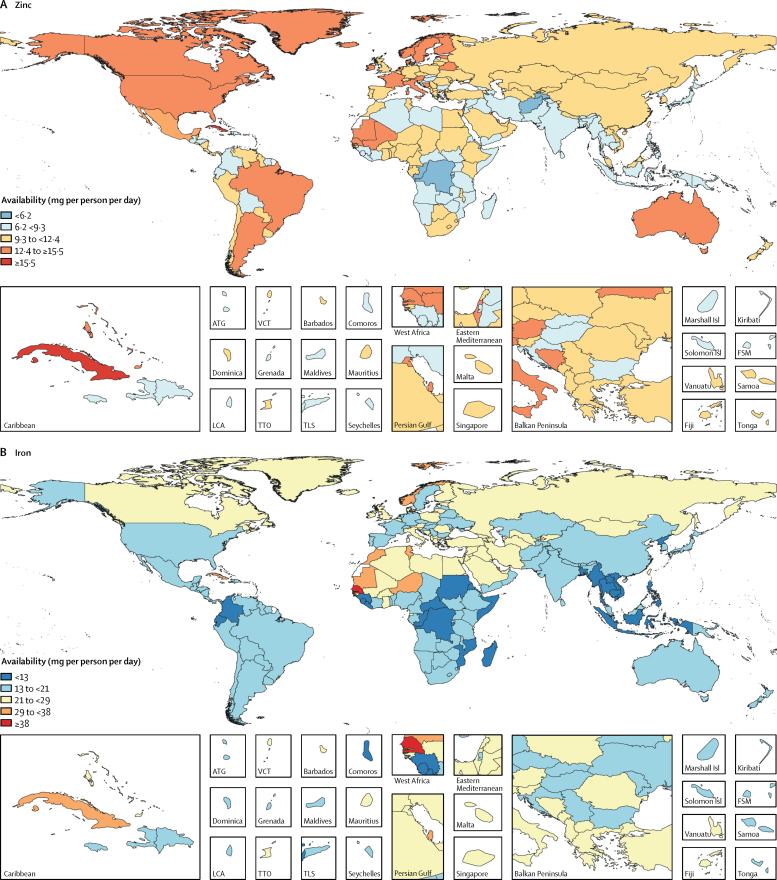

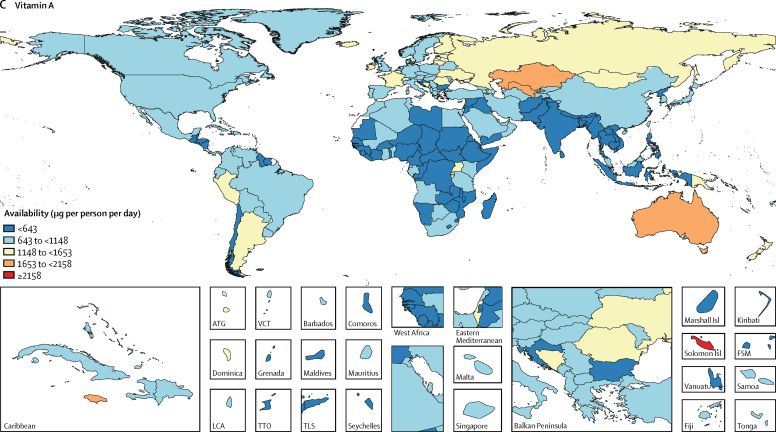
Availability of (A) zinc, (B) iron, and (C) vitamin A by country in 2013 ATG=Antigua and Barbuda. VCT=Saint Vincent and the Grenadines. LCA=Saint Lucia. TTO=Trinidad and Tobago. Isl=Islands. FSM=Federated States of Micronesia. TLS=Timor-Leste.

The availability of iron, vitamin A, and zinc varied widely across levels of development. High-SDI and upper-middle-SDI countries had higher availability of all three nutrients than did countries of lower SDI. The largest difference in the availability of these micronutrients across levels of development was for vitamin A, for which its availability in upper-middle-SDI countries (1010 μg of retinol activity equivalents [95% UI 940–1090] per person per day) was almost three times higher than that in lower-middle-SDI countries (395 μg of retinol activity equivalents [380–415] per person per day). Among the most populous countries, the greatest availabilities were in Egypt for iron (27·7 mg [26·9–28·4] per person per day), Russia for vitamin A (1200 μg of retinol activity equivalents [1120–1290] per person per day), and the USA for zinc (13·1 mg [12·9–13·2] per person per day; figure 5). The lowest availabilities of iron (9·8 mg [9·5–10·2] per person per day) and vitamin A (205 μg of retinol activity equivalents [200–210] per person per day) were in Bangladesh and the lowest availability of zinc (6·8 mg [6·6–6·9] per person per day) was in Pakistan.

The supply of the micronutrients has generally increased between 1980 and 2013 globally and across levels of development. However, the rate of increase has varied across nutrients and levels of development. Generally the highest rates of increase were in upper-middle-SDI and middle-SDI countries, while high-SDI countries had the lowest rate of increase. Among the most populous countries, the largest increases in availability were in Nigeria for iron (7·4 mg per person per day), China for vitamin A (585 μg of retinol activity equivalents per person per day), and Brazil for zinc (4·4 mg per person per day).

In our evaluation of patterns of macronutrient replacement, we found an inverse correlation between proportion of energy from carbohydrates and proportion from fats and protein. The strongest inverse correlation was between the proportion of energy from carbohydrates and the proportion from monounsaturated fat (−0·82, p<0·001), followed by the correlations between carbohydrates and saturated fat (−0·69, p<0·001) and carbohydrates and protein (−0·63, p<0·001), suggesting a substitution pattern across countries. At the same time, we observed positive correlations between protein and monounsaturated fats (0·54, p<0·001) and between monounsaturated fats and polyunsaturated fats (0·43, p<0·001).

In our validation analyses, the comparison of the estimates of the proportion of energy derived from each macronutrient between our global nutrient database and consumption data from nationally representative surveys showed a close correlation (appendix). Overall, the difference in estimated and observed proportions of energy derived from each macronutrient ranged from 1% to 4% of total energy intake. We also showed that machine-learning approaches (Random Forest and XGBoost models) could very closely predict the observed intake of macronutrient and micronutrient from the global nutrient database data (appendix). The out-of-sample correlation between predicted and observed intake of macronutrients and micronutrients was mostly greater than 0·8. For example, out-of-sample correlation between predicted and observed intake of polyunsaturated fat was 0·97 and 0·87 for Random Forest Model and XGBoost, respectively.

## Discussion

We established a database that provides a comprehensive picture of the national availability of specific macronutrients and micronutrients since 1980. Our results show that, over the past four decades, the availability of energy and all forms of macronutrients have increased globally and across levels of development. During the same period, the contributions of protein and fats to energy availability have increased, whereas the contribution of carbohydrates has decreased. We also found that, in parallel with the increase in macronutrient availability, the availability of micronutrients has increased. Our estimates showed close correlations with dietary consumption data from national representative surveys in three countries, as well as the ability to predict the level of intake of nutrients over time.

The global nutrient database provides the opportunity to answer important questions about macronutrients and micronutrients across nations. For example, the database can be used to identify countries with insufficient supplies of specific nutrients that are therefore at risk of nutrient deficiencies. Additionally, the database can be used to determine the dependency on specific foods for each nutrient in a country and over time. This information is necessary to make both agriculture and trade more nutrition-sensitive and to inform food-based interventions to prevent micronutrient deficiencies in high-risk countries. Additionally, the database can be used to assess the nutrient self-sufficiency of various countries, providing useful insights beyond the common food energy or calorie estimates.

Various groups have tried to estimate nutrient availability using FBS data.^1^, ^2^, ^4^ For example, the Global Expanded Nutrient Supply (GENuS) model used FAO production and trade data to disaggregate FBS food groups into 225 food items and estimate the supply of 23 nutrients in 152 countries. The GENuS model was validated by comparing the estimates with nutrient supply data from the US Department of Agriculture's Center for Nutrition Policy and Promotion.^1^ In the global nutrient database, we have made major improvements compared with the GENuS model. We, for the first time to our knowledge, have used more disaggregated SUA data than have previous data using FBS data, having used 394 food items to estimate the nutrient availability. We have expanded the list of the nutrients to 156 items and used spatiotemporal Gaussian process regression to estimate the nutrient supply in 195 countries and territories. Additionally, we validated our estimates by comparing them with consumption data from three countries and noted a close correlation between our estimates and consumption data.

Our results highlight important mismatches between the national supply of nutrients and the requirements of the population to achieve a healthy diet. For example, although dietary guidelines recommend decreasing intake of saturated fat to less than 7% of total energy intake, we found that the supply of saturated fats is far beyond the recommended level in many countries.^35^ Conversely, our results show that the supplies of polyunsaturated fats in many countries are not sufficient to meet the recommended level of intake (>10% of total energy intake).^11^ Additionally, our results show specific patterns of macronutrient replacement across countries. For example, although replacement of saturated fat with polyunsaturated fat has been recommended, our results provide little evidence that this type of replacement is occurring at the population level. However, we also found that, as the availability of carbohydrates decreases in a country, the availability of all types of fats, particularly monounsaturated fat and saturated fats, increases.

Our results showed a wide variation in the availability of micronutrients (eg, vitamin A) between countries and across levels of development. Generally, nutrient-specific interventions (eg, food fortification and supplementation) are regarded as effective strategies to address such types of nutrient gaps and to reduce the risk of micronutrient deficiencies in low-SDI countries.^14^ However, given the coexistence of multiple micronutrient deficiencies in low-SDI countries, concerns over the long-term health effects of common foods and nutrients used as carriers of other micronutrients in fortification programmes (eg, refined grains, processed foods, salt),^11^ and the difficulties of implementing effective fortification and supplementation programmes over a long period of time, maintaining the sustainability of these interventions might be challenging. Nutrition-sensitive interventions (eg, targeted agricultural programmes) aiming to address the root causes of micronutrient deficiencies are more promising over the long term.^36^, ^37^ The effective design and implementation of such interventions require a detailed understanding of the food sources of key nutrients at the population level and the capacity of the national food system to produce and make such foods accessible and affordable. Our global nutrient database can be useful for identifying the food sources of each nutrient in a country and inform the nutrition-sensitive programmes worldwide. Additionally, because the SUA data are collected and updated annually, our database can be used to track the progress of the countries implementing these nutrient-sensitive interventions.

Building and expanding on previous efforts,^1^, ^2^, ^3^, ^4^ we developed a model that could estimate the intake of nutrients for each age and sex group based on their availability and provide a comprehensive picture of the patterns of nutrient consumption across countries. Combined with appropriate distributional measures and cutoff points, these data can be used to quantify the prevalence of micronutrient deficiencies, estimate the number of people who are at risk of micronutrient deficiencies, and guide nutrition-specific interventions. Furthermore, this database can be used to more accurately characterise the shifts in consumption of nutrients (ie, nutrition transition) and its relationship with epidemiological transition and economic growth. Finally, the database can be used to gauge the number of plants and animals providing certain nutrients and hence the dependency of individual nutrient supplies on specific food sources. Such information will be helpful for researchers, policy makers, donors, and advocates aiming to address malnutrition globally.

Potential limitations should be taken into account when using our database. We used data from the USDA Food Composition Database to create the database, which represents the nutrient content of foods in high-SDI countries. Additionally, the inedible proportion of each food was considered to be the same across countries and over time. Given that nutrient content of foods and their edible proportion might vary across countries and over time, the availability of some nutrients in our database might be underestimated or overestimated. We did not incorporate data on existing fortification programmes into our estimates of nutrient availability. Although such data can be useful in characterising the consumption of specific nutrients in some populations more accurately, including these data could underestimate the real gap in food-based supply of nutrients. We did not take into account processing of foods at the retail level or at the consumer level. Therefore, our database, in its current form, cannot be used to estimate the availability or consumption of specific nutrients that are added during food processing (eg, sodium or trans fats). In our validation analysis, we compared our results with national nutrition surveys from only three countries and the accuracy of our estimates in other countries (eg, those in Africa) needs to be evaluated. As with FAO's FBS and SUA data, our estimates of nutrient availability, in their current format, do not reflect consumption levels. Similarly, our estimates do not provide any information about the distribution of nutrients within a country or the level of access to nutrients in each country.

In conclusion, the global nutrient database provides a comprehensive picture of the availability of a wide range of nutrients across countries and over time. In future it could be used to inform evidence-based nutrition-sensitive interventions to end all forms of malnutrition.
