# Supplementary material for: The Global Nutrient Database: availability of macronutrients and micronutrients in 195 countries from 1980 to 2013
Source: Lancet Planet Health. 2018 Aug;2(8):e353–68. doi: 10.1016/S2542-5196(18)30170-0 (PMC6076406; doi:10.1016/S2542-5196(18)30170-0)
Supplement: Supplementary appendix [file mmc1.pdf]

THE LANCET
Planetary Health

Supplementary appendix

This appendix formed part of the original submission and has been peer reviewed. We post it as supplied by the authors.

Supplement to: Schmidhuber J, Sur P, Fay K, et al. The Global Nutrient Database: a systematic assessment of the availability of macronutrients and micronutrients in 195 countries from 1980 to 2013. *Lancet Planet Health* 2018; **2:** e353–68.

Global Nutrient Database

Supplemental Table 1. List of the nutrients included in the Global Nutrient Database	2
Supplemental Table 2. List of the food and agricultural commodities included in Supply and Utilization Accounts and their corresponding refuse factor	3
Supplemental Table 3. Performance of Random Forest models for predicting the intake of selected nutrients	17
Spatio-temporal Gaussian Process Regression	18
Supplemental Figure 1.Relationship between the percent of energy from different macronutrients	19
Supplemental Figure 2. Comparison of the percent of energy from macronutrients in the Global Nutrient Database and the United States National Health and Nutrition Examination Survey (NHANES)	34

Supplemental Table 1. List of the nutrients estimated in the Global Nutrient Database

Alanine	Fatty acids 20:00	Pantothenic acid
Arginine	Fatty acids 20:01	Phenylalanine
Ash	Fatty acids 20:02 n-6 c,c	Phosphorus, P
Aspartic acid	Fatty acids 20:03 n-3	Phytosterols
Betaine 2075	Fatty acids 20:03 n-6	Potassium, K
Beta-sitosterol	Fatty acids 20:03 undifferentiated	Proline
Calcium, Ca	Fatty acids 20:04 n-6	Protein
Campesterol	Fatty acids 20:04 undifferentiated	Retinol
Carbohydrate, by difference	Fatty acids 20:05 n-3 (EPA)	Riboflavin
Carotene, alpha	Fatty acids 21:05	Selenium, Se
Carotene, beta	Fatty acids 22:00	Serine
Cholesterol	Fatty acids 22:01 c	Share of calories from Carbohydrates %
Choline, total	Fatty acids 22:01 t	Share of calories from Lipids %
Copper, Cu	Fatty acids 22:01 undifferentiated	Share of calories from Monounsaturated F
Cryptoxanthin, beta	Fatty acids 22:04	Share of calories from Polyunsaturated F
Cystine	Fatty acids 22:05 n-3 (DPA)	Share of calories from Proteins %
Dihydrophylloquinone	Fatty acids 22:06 n-3 (DHA)	Share of calories from Saturated Fats %
Energy	Fatty acids 24:00:00	Share of FAO sugar in FAO calories %
Energy	Fatty acids 24:01:00 c	Sodium, Na
FAO calories from sugar in Kcal/person/day	Fatty acids, total monounsaturated	Starch
FAO DES (Kcal/p/d)	Fatty acids, total polyunsaturated	Stigmasterol
Fatty acids 04:00	Fatty acids, total saturated	Sucrose
Fatty acids 06:00	Fatty acids, total trans 3243	Sugar
Fatty acids 08:00	Fatty acids, total transmonoenoic	Sugars, total
Fatty acids 12:00	Fatty acids, total transpolyenoic	Sweeteners
Fatty acids 13:00	Fatty acids10:0	Thiamin
Fatty acids 14:00	Fiber, total dietary	Threonine
Fatty acids 14:01	Fluoride, F 532	Tocopherol, beta
Fatty acids 15:00	Folate, DFE	Tocopherol, delta
Fatty acids 15:01	Folate, food	Tocopherol, gamma
Fatty acids 16:00	Folate, total	Tocotrienol, alpha
Fatty acids 16:01 c	Folic acid	Tocotrienol, beta
Fatty acids 16:01 t	Fructose	Tocotrienol, delta
Fatty acids 16:01 undifferentiated	Galactose	Tocotrienol, gamma
Fatty acids 17:00	Glucose (dextrose)	Total lipid (fat)
Fatty acids 17:01	Glutamic acid	Tryptophan
Fatty acids 18:00	Glycine	Tyrosine
Fatty acids 18:01 c	Histidine	Valine
Fatty acids 18:01 t	Hydroxyproline	Vitamin A, IU
Fatty acids 18:01 undifferentiated	Iron, Fe	Vitamin A, RAE
Fatty acids 18:02 CLAs	Isoleucine	Vitamin B-12
Fatty acids 18:02 i 60	Lactose	Vitamin B-12, added
Fatty acids 18:02 n-6 c,c	Leucine	Vitamin B-6
Fatty acids 18:02 t not	Lutein + zeaxanthin	Vitamin D
Fatty acids 18:02 t,t	Lycopene	Vitamin D (D2 + D3)
Fatty acids 18:02 undifferentiated	Lysine	Vitamin D2 (ergocalciferol)
Fatty acids 18:03 n-3 c,c,c	Magnesium, Mg	Vitamin D3 (cholecalciferol)
Fatty acids 18:03 n-6 c,c,c	Maltose	Vitamin E (alphatocopherol)
Fatty acids 18:03 undifferentiated	Manganese, Mn	Vitamin E, added
Fatty acids 18:04	Menaquinone-4	Vitamin K (phylloquinone)
Fatty acids 18:1-11 t (18:1t)	Methionine	Water
Fatty acids 18:3i	Niacin	Zinc, Zn

Supplemental Table 2. List of the food and agricultural commodities included in Supply and Utilization Accounts and their corresponding refuse factor

Food Item	Refuse factor (%)
Almonds	60
Almonds Shelled	0
Animal Oils and Fats nes	0
Anise, Badian, Fennel	0
Applejuice Concentrated	0
Applejuice Single Streng	0
Apples	8
Apricots	7
Areca Nuts (Betel)	54
Artichokes	60
Asparagus	47
Avocados	26
Bacon-Ham of Pigs	3
Bambara Beans	47
Bananas	36
Barley	0
Barley Flour and Grits	0
Barley, Pearled	0
Beans, Dry	0
Beans, Green	56
Beef and Veal	19
Beef and Veal,Boneless	2
Beef Dried Salt Smoked	0
Beef Preparations	0
Beer of Barley	0
Beer of Millet	0

Beer of Sorghum	0
Berries nes	0
Beverages Dist Alcoholic	0
Beverages Non-Alcoholic	0
Blueberries	2
Bran of Wheat	0
Brazil Nuts	52
Brazilnuts Shelled	52
Bread	0
Breakfast Cereals	0
Broad Beans, Dry	0
Broad Beans, Green	3
Buckwheat	0
Buffalo Meat	0
Buffalo Milk	0
Bulgur, Wholemeal	0
Butter of Cow Milk	0
Butter of Karite Nuts	0
Butter+Ghee (Sheep Milk)	0
Butterm,Curdl,Acid.Milk	0
Cabbages	12
Camel Milk	0
Canned Mushrooms	0
Cantaloupes&oth Melons	49
Carobs	0
Carrots	11
Casein	0
Cashew Nuts	0
Cashew Nuts Shelled	0
Cashewapple	40

Cassava	26
Cassava Dried	0
Cassava Starch	0
Cassava Tapioca	0
Cauliflower	61
Cereal Prep nes	0
Cereals nes	0
Cheese (Skim Cow Milk)	0
Cheese (Whole Cow Milk)	0
Cheese of Buffalo Milk	0
Cheese of Goat Milk	0
Cheese of Sheep Milk	0
Cherries	10
Chestnuts	26
Chicken Meat	31
Chick-Peas	0
Chicory Roots	18
Chillies&Peppers, Green	27
Chocolate Products nes	0
Cinnamon (Canella)	0
Citrus Fruit nes	27
Citrusjuice Concentrated	0
Citrusjuice Single-Stren	0
Cloves, Whole+Stems	0
Cocoa Beans	0
Cocoa Butter	0
Cocoa Paste	0
Cocoa Powder and Cake	0
Coconuts	48
Coconuts, Dessicated	0

Coffee Extracts	0
Coffee Roasted	0
Coffee Subst Cont Coffee	0
Coffee, Green	0
Copra	0
Cow Milk, Whole, Fresh	0
Cow Peas, Dry	0
Cranberries	5
Cream, Fresh	0
Cucumbers and Gherkins	27
Currants	2
Dates	10
Dried Mushrooms	0
Dry Apricots	0
Dry Buttermilk	0
Dry Skim Cow Milk	0
Dry Whey	0
Dry Whole Cow Milk	0
Duck Meat	28
Egg Albumine	0
Eggplants	19
Eggs Dry Whole Yolks Hen	0
Eggs Liquid Hen	12
Eggs, excluding Hen	13
Extract Tea,Mate, Prep.	0
Fat Liver Prep(Foie Gras	0
Fat of Buffalo	0
Fat of Camels	0
Fat of Cattle	0
Fat of Goats	0

Fat of Pigs	0
Fat of Poultry	0
Fat of Poultry Rendered	0
Fat of Sheep	0
Fat Preparations nes	0
Ferm. Beverages Exc Wine	0
Figs	1
Figs, Dried	1
Flour of Buckwheat	0
Flour of Cassava	0
Flour of Cereals	0
Flour of Fonio	0
Flour of Fruit	0
Flour of Maize	0
Flour of Millet	0
Flour of Mustard	0
Flour of Potatoes	0
Flour of Pulses	0
Flour of Roots and Tuber	0
Flour of Rye	0
Flour of Sorghum	0
Flour of Triticale	0
Flour of Wheat	0
Flour/Meal of Oilseeds	0
Food Prep.Flour,Malt Ext	0
Food Prepared nes	8
Fructose Chemically Pure	0
Fruit Dried nes	6
Fruit Fresh nes	40
Fruit Juice nes	0

Fruit Prepared nes	0
Fruit Tropical Dried nes	10
Fruit Tropical Fresh nes	48
Fruit,Nut,Peel,Sugar Prs	0
Game Meat	19
Garlic	13
Germ of Maize	0
Germ of Wheat	0
Ghee (From Buffalo Milk)	0
Ghee (From Cow Milk)	0
Ginger	0
Glucose and Dextrose	0
Goat Meat	0
Goat Milk	0
Goose Meat	19
Grape Juice	0
Grapefruit and Pomelos	50
Grapefruitjuice Concentr	0
Grapefruitjuice Sing-Str	0
Grapes	42
Green Corn (Maize)	64
Groundnuts in Shell	47
Groundnuts Shelled	0
Hazelnuts (Filberts)	54
Hazelnuts Shelled	0
Hen Eggs	12
Homogen.Cooked Fruit Pre	0
Homogenized Veget. Prep.	0
Honey	0
Horsemeat	19

Ice Cream and Edible Ice	0
Infant Food	0
Juice of Vegetables nes	0
Karite Nuts (Sheanuts)	39
Kiwi Fruit	14
Kolanuts	23
Lactose	0
Lard	0
Leeks and Oth.Alliac.Veg	56
Lemonjuice Concentrated	0
Lemonjuice Single-Streng	0
Lemons and Limes	47
Lentils	0
Lettuce	5
Liquid Margarine	0
Liver Preparations	0
Lupins	0
Macaroni	0
Maize	64
Maize Gluten	0
Malt Extracts	0
Malt of Barley	0
Maltose Chemically Pure	0
Mango Juice	0
Mango Pulp	31
Mangoes	31
Maple Sugar and Syrups	0
Margarine + Shortening	0
Mate	0
Meat Canned Chicken	0

Meat Extracts	0
Meat nes	31
Meat of Asses	19
Meat of Camels	19
Meat of Pigeon Oth.Birds	23
Meat Preparations Pigs	24
Meat Prepared nes	0
Meat, Dried, nes	0
Melonseed	63
Milled Paddy Rice	0
Milled/Husked Rice	0
Millet	0
Mixes and Doughs	0
Molasses	0
Mushrooms	3
Must of Grapes	0
Mustard Seed	0
Mutton and Lamb	0
Nutmeg, Mace, Cardamons	0
Nuts nes	69
Oats	0
Oats, Rolled	0
Offals Liver Ducks	0
Offals Liver Geese	0
Offals Liver of Chickens	0
Offals Liver Turkeys	0
Offals nes	0
Offals of Buffalo,Edible	0
Offals of Camel, Edible	0
Offals of Cattle, Edible	0

Offals of Goats, Edible	0
Offals of Horse	0
Offals of Pigs, Edible	0
Offals of Sheep, Edible	0
Oil of Coconuts	0
Oil of Cotton Seed	0
Oil of Groundnuts	0
Oil of Linseed	0
Oil of Maize	0
Oil of Mustard Seed	0
Oil of Olive	0
Oil of Olive Residues	0
Oil of Palm	0
Oil of Palm Kernels	0
Oil of Rapeseed	0
Oil of Rice Bran	0
Oil of Safflower	0
Oil of Sesame Seed	0
Oil of Soya Beans	0
Oil of Sunflower Seed	0
Oil of Veget Origin nes	0
Oils Hydrogenated	0
Oilseeds nes	0
Okra	14
Olives	22
Olives, Preserved	0
Onions, Dry	10
Onions+Shallots, Green	12
Oranges	27
Oranjuice Concentrated	0

Oranjuice Single-Strengt	0
Other Fructose and Syrup	0
Palm Kernels	0
Papayas	33
Pastry	0
Peaches and Nectarines	13
Peanut Butter	0
Pears	8
Peas, Dry	0
Peas, Green	69
Peeled Tomatoes	0
Pepper,White/Long/Black	0
Persimmons	16
Pig Butcher Fat	0
Pigeon Peas	0
Pigmeat	25
Pimento, Allspice	0
Pineapplejuice Concentr.	0
Pineapplejuice Sing-Stre	0
Pineapples	48
Pineapples, Canned	0
Pistachios	50
Plantains	35
Plums	6
Plums, Dried (Prunes)	13
Pop Corn	0
Poppy Seed	0
Pork	30
Potato Starch	0
Potato Tapioca	0

Potatoes	15
Potatoes, frozen	0
Prepared Groundnuts	0
Preprd Nuts(Excl.Grnuts)	0
Processed Cheese	0
Prod.Of Nat.Milk Constit	0
Pulses nes	2
Pumpkins, Squash, Gourds	30
Quinces	39
Quinoa	0
Rabbit Meat	21
Raisins	0
Rapeseed	46
Raspberries	4
Reconstituted Milk	0
Rice Fermented Beverages	0
Rice Flour	0
Rice Gluten	0
Rice, Broken	0
Rice, Husked	0
Rice, Paddy	35
Rice, Starch	0
Roots and Tubers Dried	0
Roots and Tubers nes	15
Rye	0
Safflower Seed	49
Sausages Beef and Veal	0
Sausages Pig Meat	0
Sesame Seed	0
Sheep Milk	0

Skim Milk of Cows	0
Skim Milk, Condensed	0
Skim Milk, Evaporated	0
Skim Sheep Milk	0
Snails Not Sea Snails	31
Sorghum	0
Sour Cherries	10
Soya Curd	0
Soya Sauce	0
Soybeans	0
Spices nes	0
Spinach	28
Starch of Maize	0
Stone Fruit nes, Fresh	13
Strawberries	6
String Beans	12
Sugar and Syrups nes	0
Sugar Cane	0
Sugar Confectionery	0
Sugar Crops nes	0
Sugar non Centrifugal	0
Sugar Refined	0
Sugars Flavoured	0
Sunflower Seed	46
Sweet Corn Frozen	45
Sweet Corn Prep. or Pres	0
Sweet Potatoes	28
Tallow	0
Tang.Mand.Clement.Satsma	28
Tangerine Juice	0

Taro (Coco Yam)	14
Tea	0
Tea nes	0
Tomato Paste	0
Tomatoes	9
Tomatojuice Concentrated	0
Tomatojuice Single-Stren	0
Turkey Meat	21
Vanilla	0
Veg Prod Fresh or Dried	9
Veg. in Temp Preservativ	0
Veg.Prep or Pres.Frozen	0
Vegetables Canned nes	0
Vegetables Dehydrated	0
Vegetables Fresh nes	71
Vegetables Frozen	0
Vegetables Pr by Vinegar	0
Vegetables Prepared nes	0
Vermouths and Similar	0
Vetches	0
Wafers	0
Walnuts	55
Walnuts Shelled	55
Watermelons	48
Waters,Ice, etc.	0
Wheat	0
Wheat Gluten	0
Wheat Starch	0
Whey Cheese	0
Whey, Condensed	0

Whey, Fresh	0
Whole Milk, Evaporated	0
Whole Milk,Condensed	0
Wine	0
Yams	14
Yautia (Cocoyam)	14
Yoghurt	0
Yogurt Concentr.Or Not	0

Supplemental Table 3. Performance of Random Forest models for predicting the intake of selected nutrients

Nutrient	Out-of-sample root mean-square error	Out-of-sample Pearson correlation
Calcium (g/day)	0.084	0.919
Fiber (g/day)	2.169	0.942
Polyunsaturated fat (% energy/day)	0.005	0.968
Saturated fat (% energy/day)	0.01	0.929
Zinc (g/day)	0.002	0.835

Spatio-temporal Gaussian Process Regression

We use a spatio-temporal Gaussian process regression to estimate the full time series of national availability of nutrients. This modelling approach has been described in detail elsewhere. Briefly, *we* estimated the mean availability of each nutrient in country (c) at time (t) using the equation below:

$$\log(\mathrm{nutrient}_{\mathrm{c,t}}) = \mathrm{g_c(t)} + \epsilon_{\mathrm{c,t}}$$

$$\epsilon_{c,t} \sim Normal(0, \sigma_p^2\ )$$

$$g_c\ (t) \sim GP\left(m_c(t), Cov(g_c(t))\right)$$

σ_p^2 represents the error variance, which is composed of the squared standard error of the observed data point as well as the prediction errors from the adjustment, if applicable. We used an empirical mean prior, which was the output of the first-stage linear model plus the smoothed residuals. The equation is shown below:

$$m_c(t) = X_c\beta + h(r_{c,t})$$

where $X\beta$ is the summation of the components of a the linear regression model, including the intercept and the product of covariates with their corresponding fixed effect coefficients. The second part of the equation, $h(r_{c,t})$, is a smoothing function for the residuals, $r_{c,t}$, derived from the linear model. For the covariance function, we used the Matern covariance function, equation below.

$$M(t,t') = \sigma^2 \frac{2^{1-v}}{\Gamma(\mathrm{v})} \left(\frac{d(t,t')\sqrt{2v}}{l}\right)^v K_v\left(\frac{d(t,t')\sqrt{2v}}{l}\right)$$

where $d(\cdot)$is a distance function; σ^2, v, l, and K_v are hyperparameters of the covariance function—specifically σ^2 is the marginal variance, v is the smoothness parameter that defines the differentiability of the function, l is the length scale, which roughly defines the distance between which two points become uncorrelated, and K_v is the Bessel function. Based on previous analyses, we approximated σ^2 by $MADN(r'_{c,t})$, which is the normalized absolute deviation of the residuals from the smoothing step by region and used the parameter specifications $v = 2$ and $l = 15$. Based on the specification above, to predict the time series of availability of $nutrient_{c,t_*}$, for country *c for* time t_*, we integrated over $g_c(t_*)$ to obtain the following:

$$log(intake_{c,t_*}) \sim N\left(m_c(t_*), \sigma_p^2 I + Cov(g_c(t_*))\right)$$

From this derivation, we generated the mean estimates as well as the uncertainty intervals. The analysis was implemented though PyMC package in Python. Random draws of 1,000 samples was obtained from the distributions above for every country. The final estimated availability for each country is the mean of the draws. In addition, uncertainty intervals were obtained by taking the 2.5 and 97.5 percentiles of the samples.

Supplemental Figure 1.Relationship of percent of energy from different macronutrients.

Pearson correlation: 0.159 in 1980; 0.146 in 2013

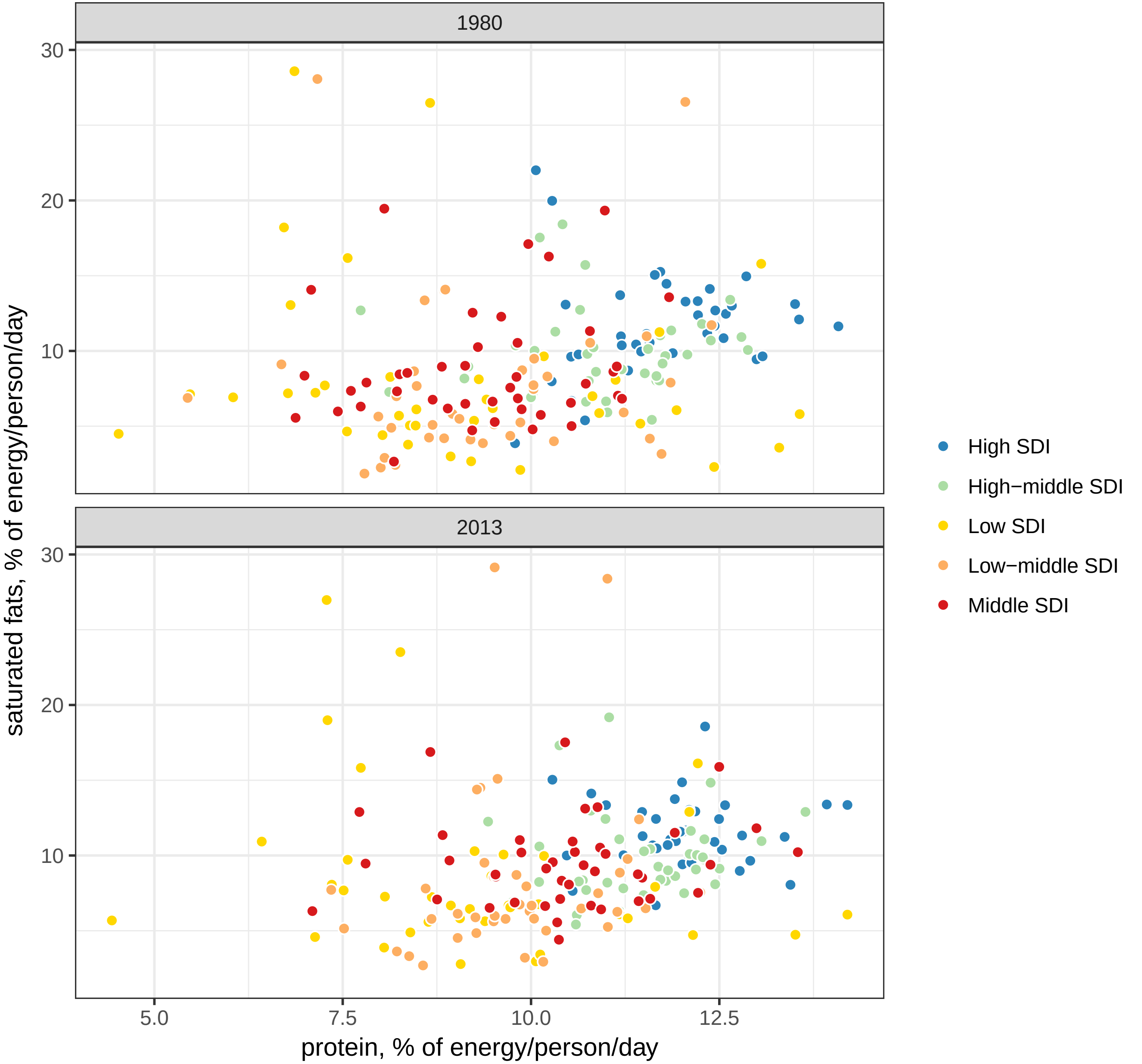

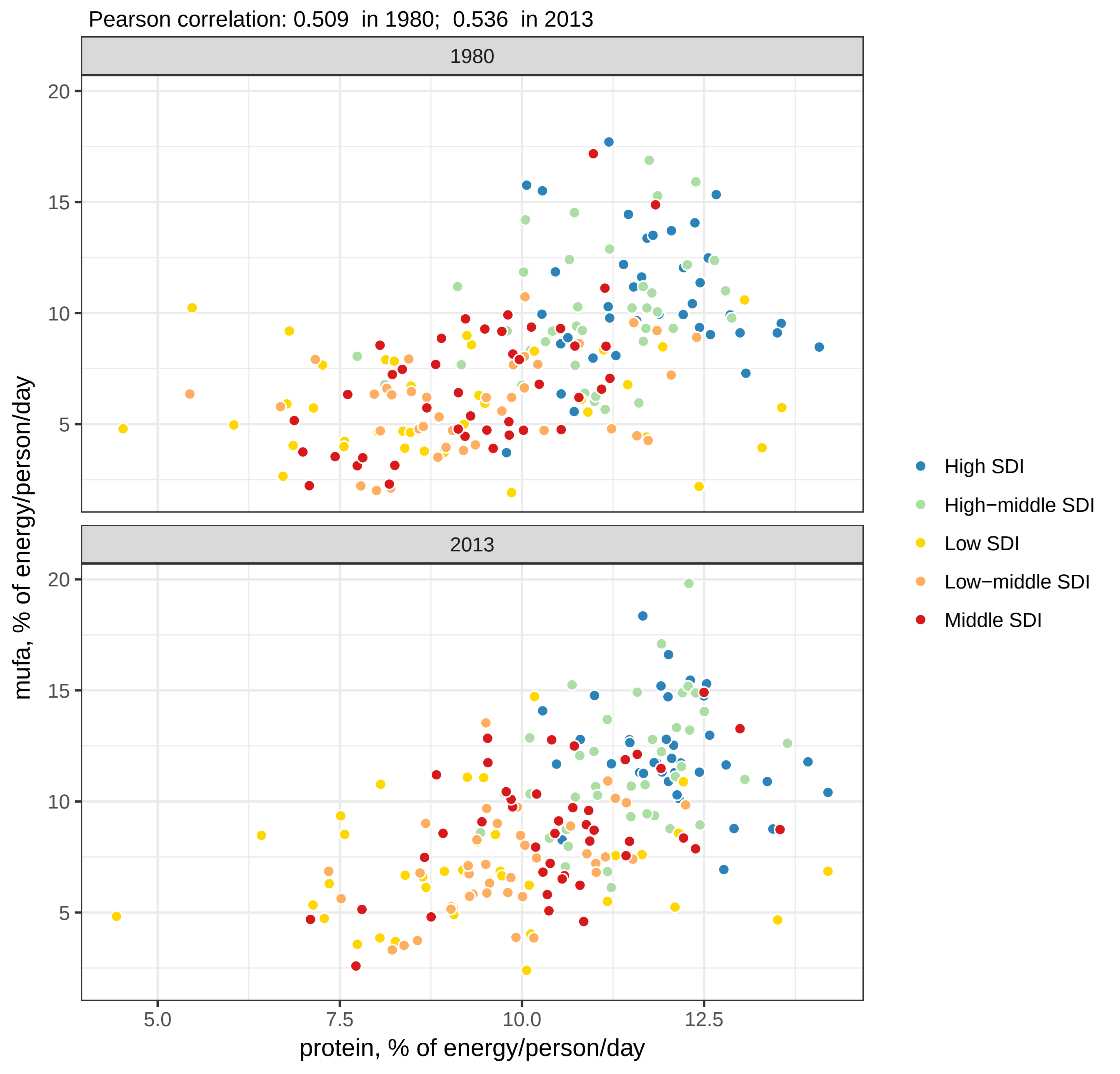

Pearson correlation: 0.509 in 1980; 0.536 in 2013
1980
2013
mufa, % of energy/person/day
protein, % of energy/person/day
20
15
10
5
5.0
7.5
10.0
12.5
High SDI
High−middle SDI
Low SDI
Low−middle SDI
Middle SDI

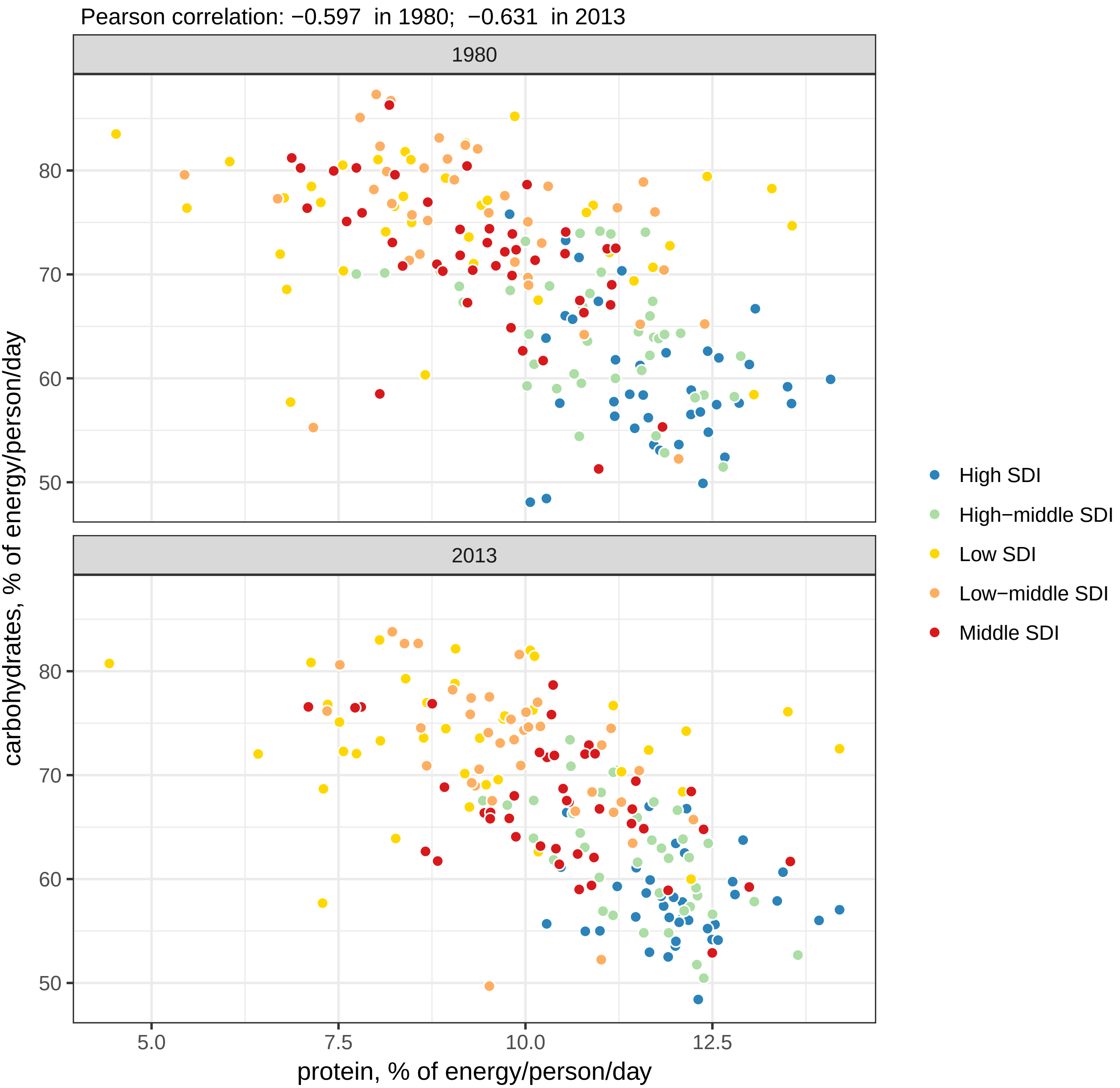
Pearson correlation: −0.597 in 1980; −0.631 in 2013
1980
2013
carbohydrates, % of energy/person/day
protein, % of energy/person/day
50
60
70
80
5.0
7.5
10.0
12.5
High SDI
High−middle SDI
Low SDI
Low−middle SDI
Middle SDI

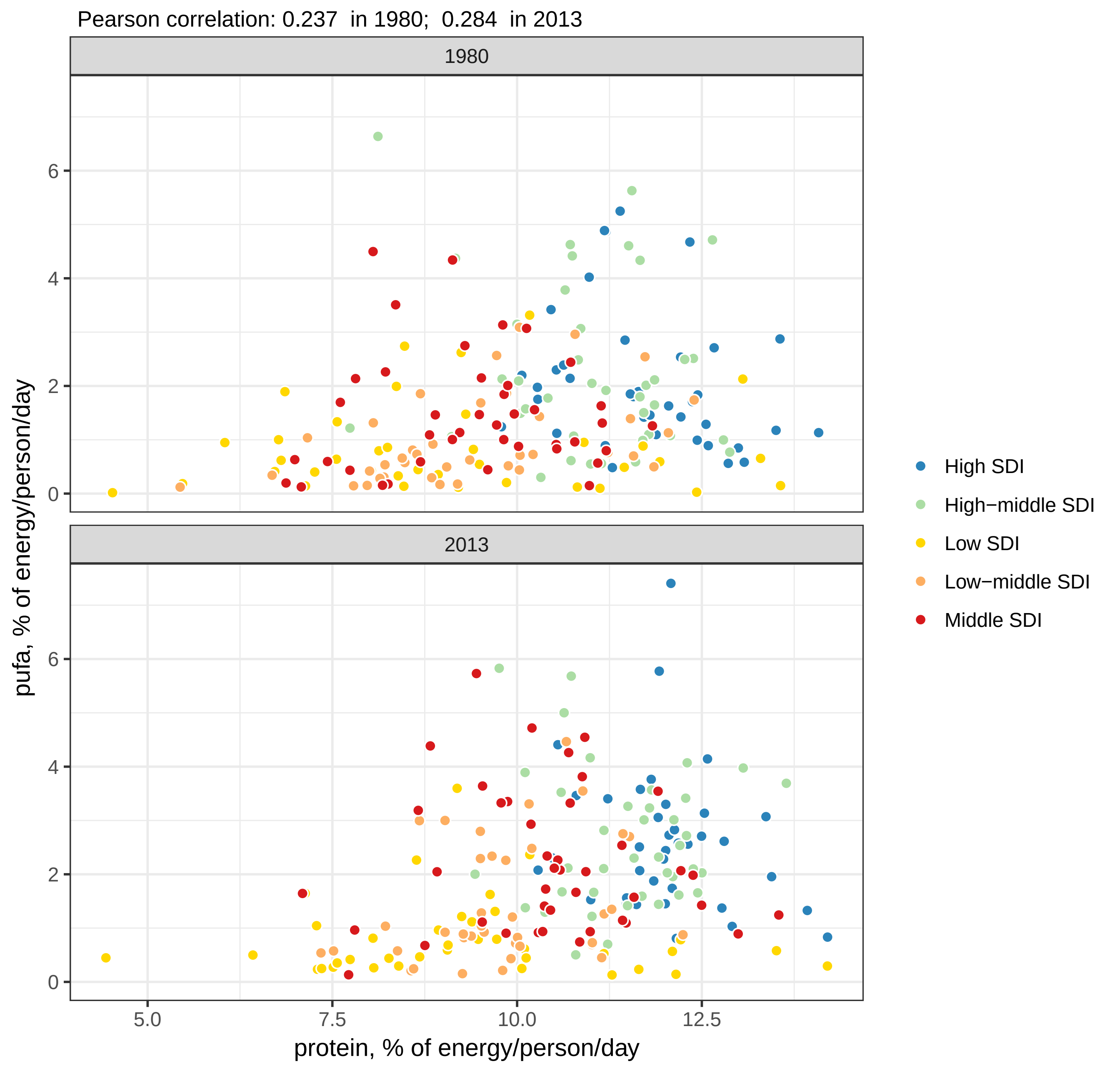

Pearson correlation: 0.237 in 1980; 0.284 in 2013
1980
2013
pufa, % of energy/person/day
protein, % of energy/person/day
0
2
4
6
5.0
7.5
10.0
12.5
High SDI
High−middle SDI
Low SDI
Low−middle SDI
Middle SDI

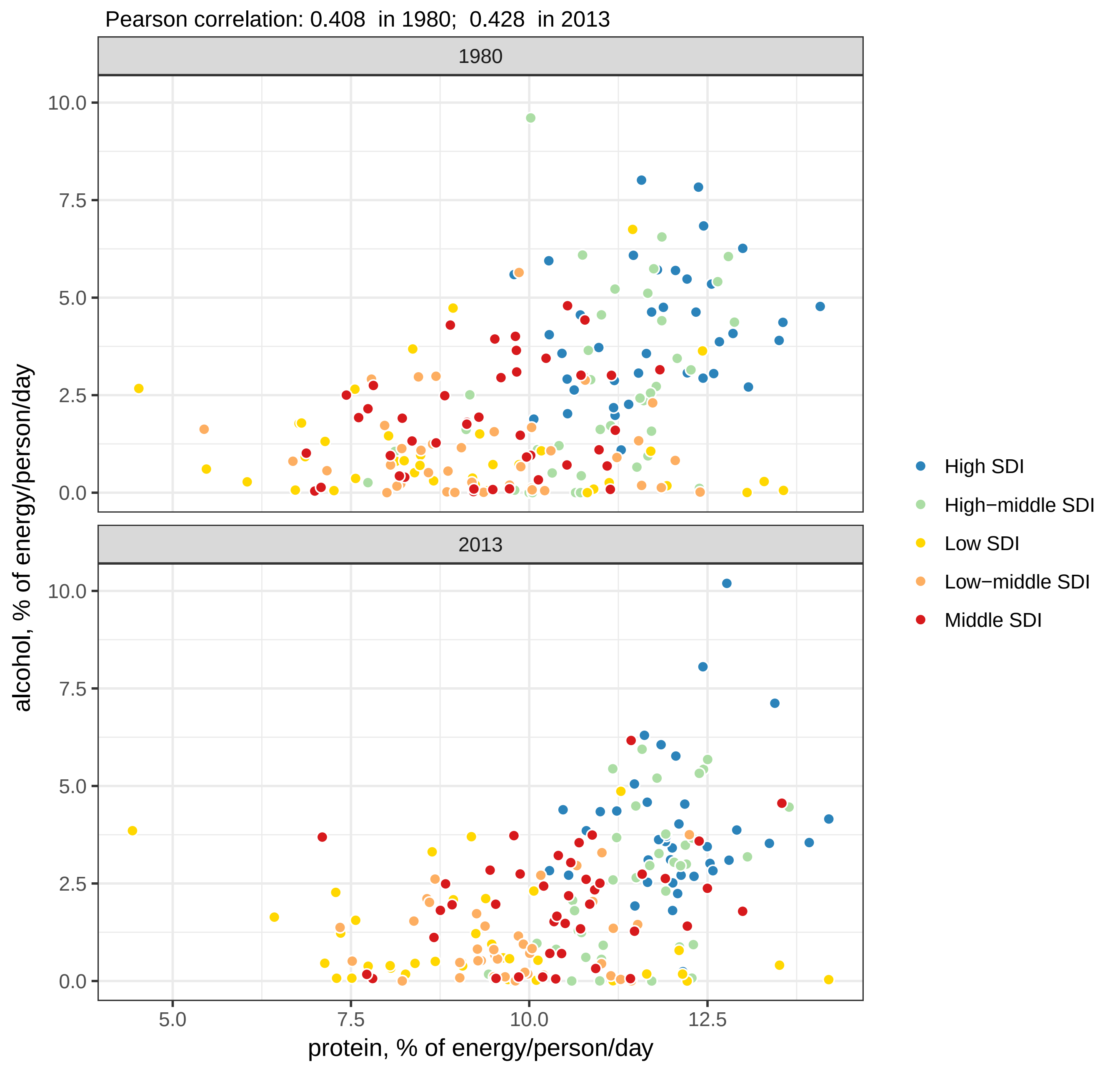
Pearson correlation: 0.408 in 1980; 0.428 in 2013
1980
2013
alcohol, % of energy/person/day
protein, % of energy/person/day
0.0
2.5
5.0
7.5
10.0
12.5
High SDI
High−middle SDI
Low SDI
Low−middle SDI
Middle SDI

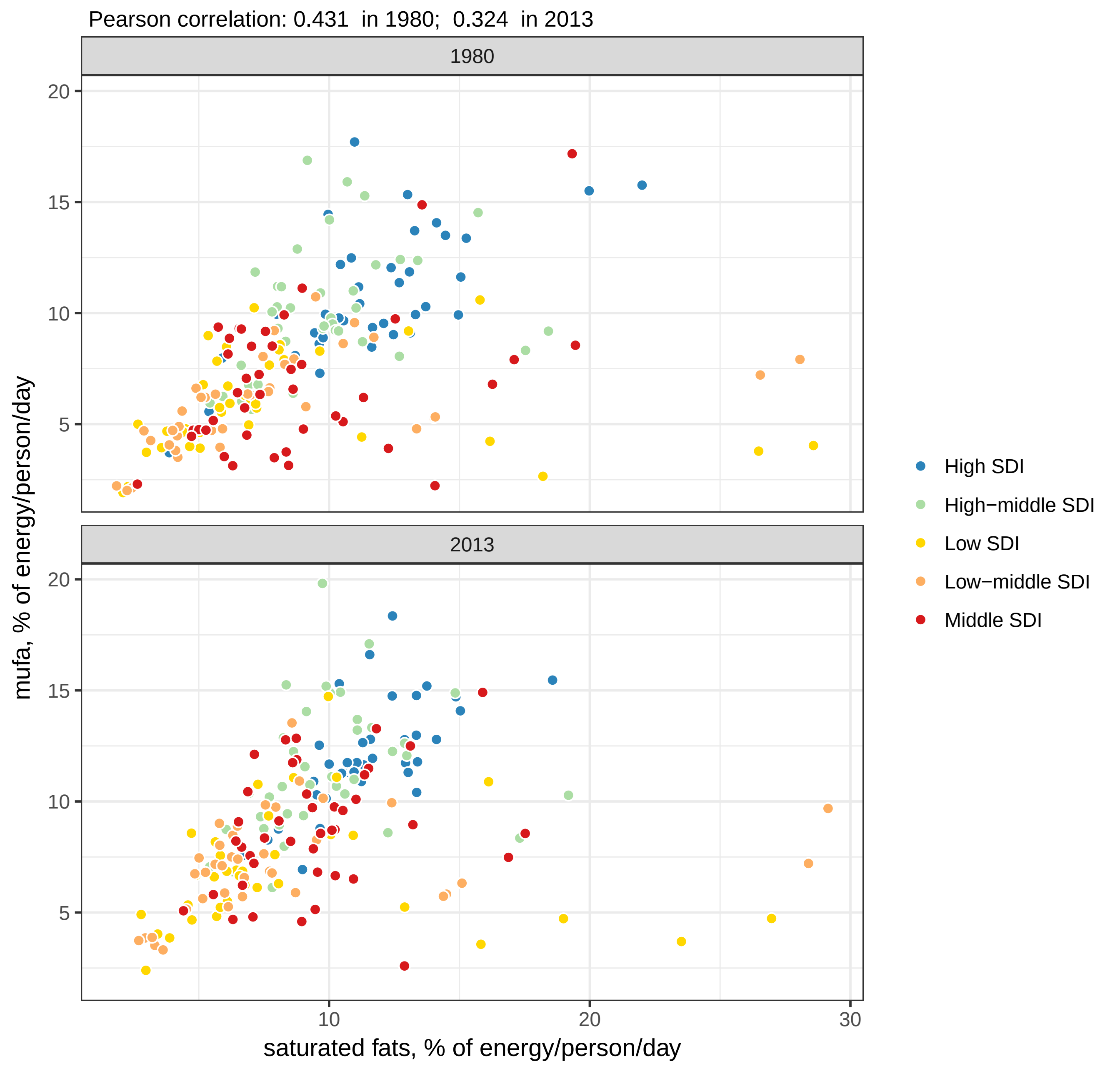

Pearson correlation: 0.431 in 1980; 0.324 in 2013
1980
2013
mufa, % of energy/person/day
saturated fats, % of energy/person/day
20
15
10
5
20
15
10
5
10
20
30
High SDI
High−middle SDI
Low SDI
Low−middle SDI
Middle SDI

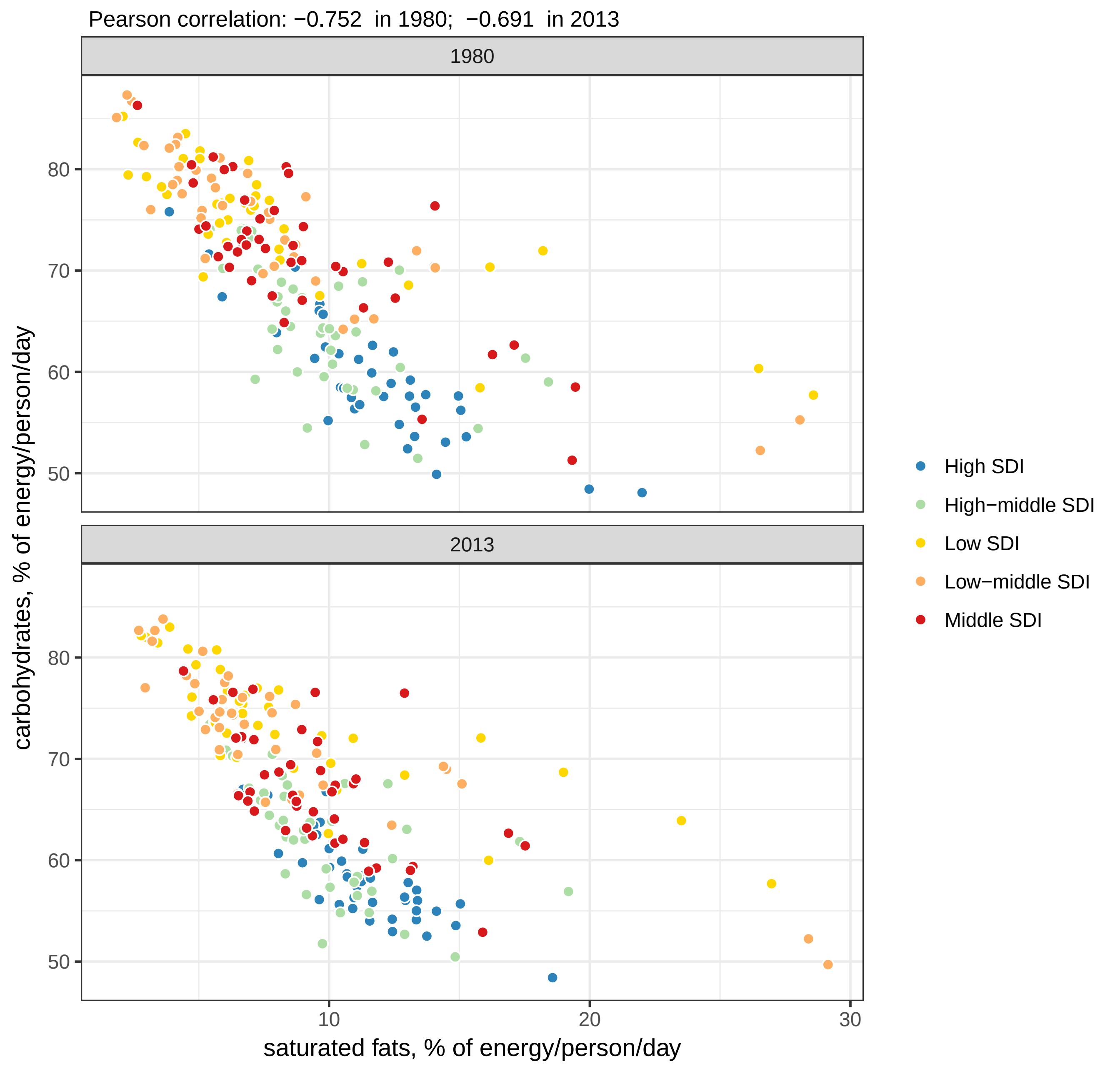

Pearson correlation: −0.752 in 1980; −0.691 in 2013
1980
2013
carbohydrates, % of energy/person/day
saturated fats, % of energy/person/day
80
70
60
50
10
20
30
High SDI
High−middle SDI
Low SDI
Low−middle SDI
Middle SDI

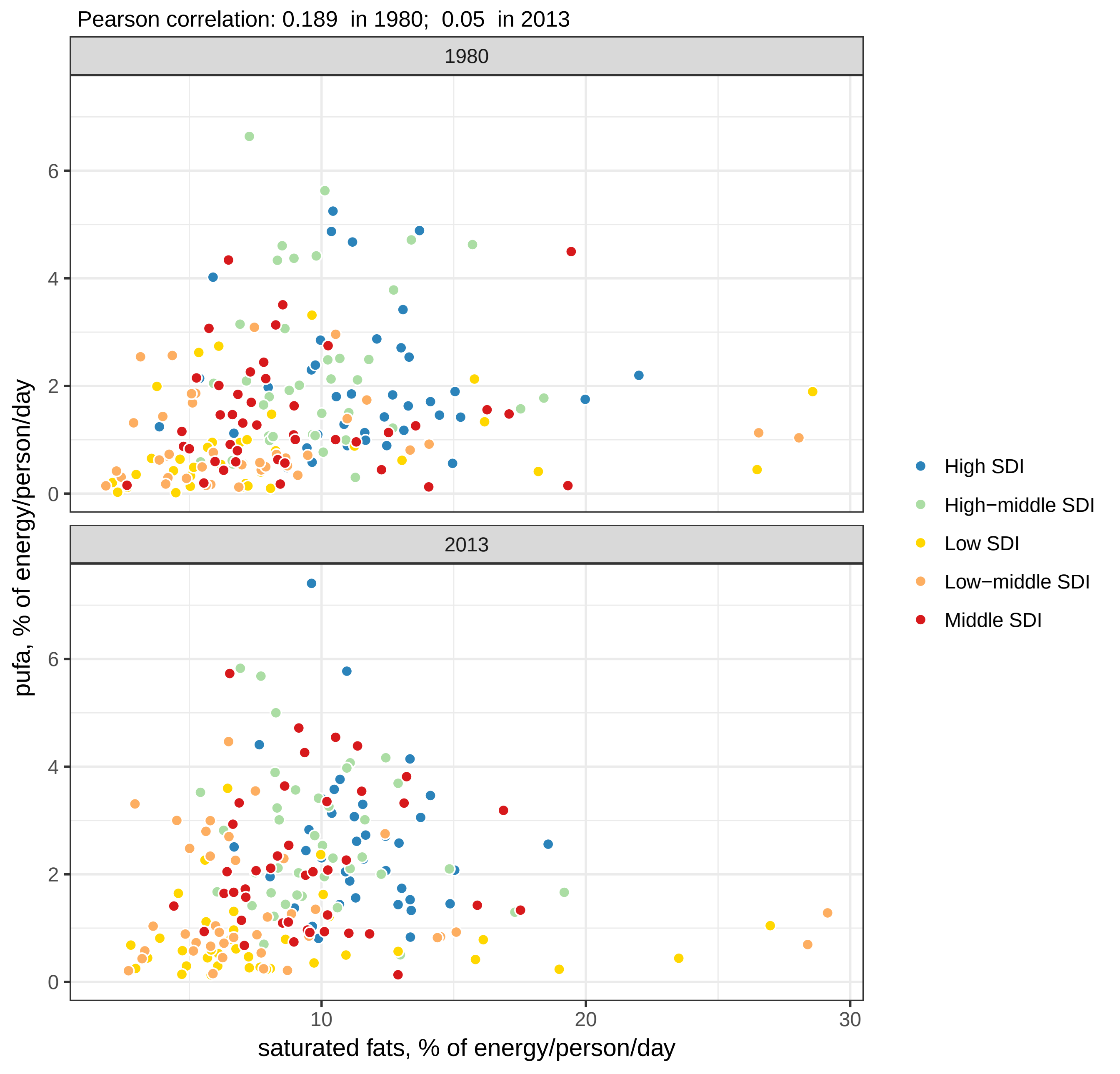

Pearson correlation: 0.189 in 1980; 0.05 in 2013
1980
2013
pufa, % of energy/person/day
saturated fats, % of energy/person/day
0
2
4
6
10
20
30
High SDI
High−middle SDI
Low SDI
Low−middle SDI
Middle SDI

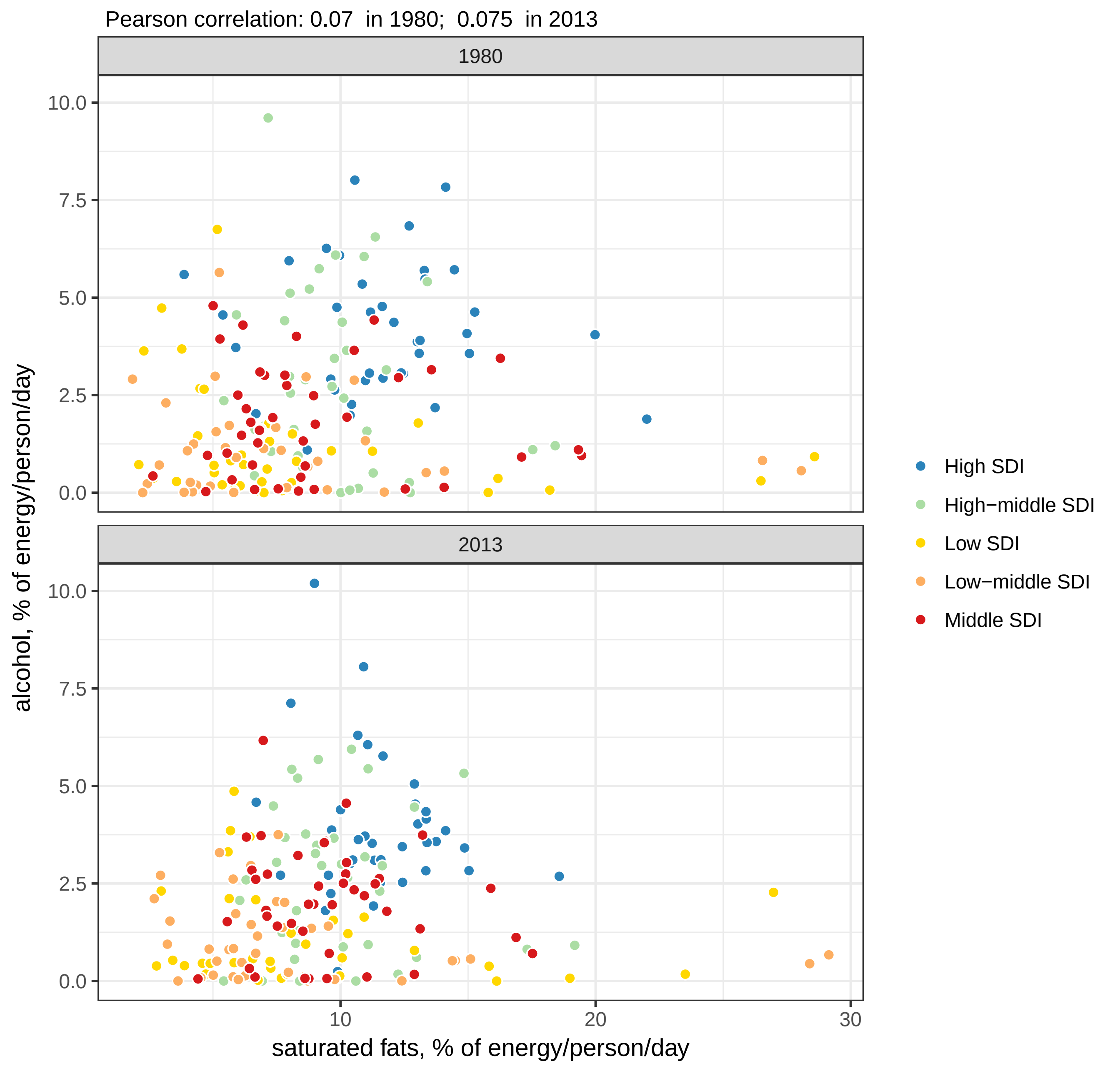
Pearson correlation: 0.07 in 1980; 0.075 in 2013
1980
2013
alcohol, % of energy/person/day
saturated fats, % of energy/person/day
0.0
2.5
5.0
7.5
10.0
10
20
30
High SDI
High−middle SDI
Low SDI
Low−middle SDI
Middle SDI

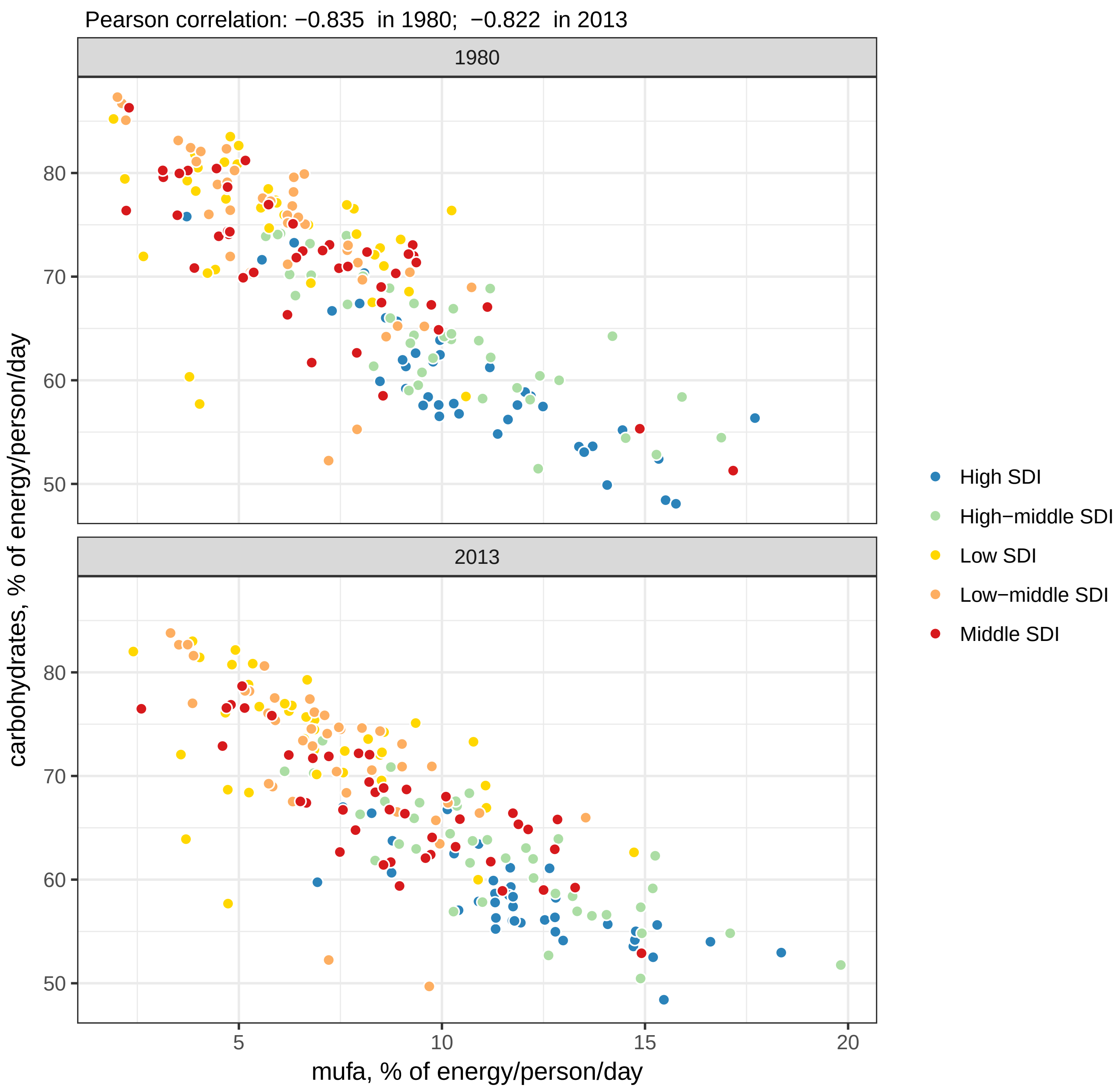

Pearson correlation: −0.835 in 1980; −0.822 in 2013
1980
2013
carbohydrates, % of energy/person/day
mufa, % of energy/person/day
50
60
70
80
5
10
15
20
High SDI
High−middle SDI
Low SDI
Low−middle SDI
Middle SDI

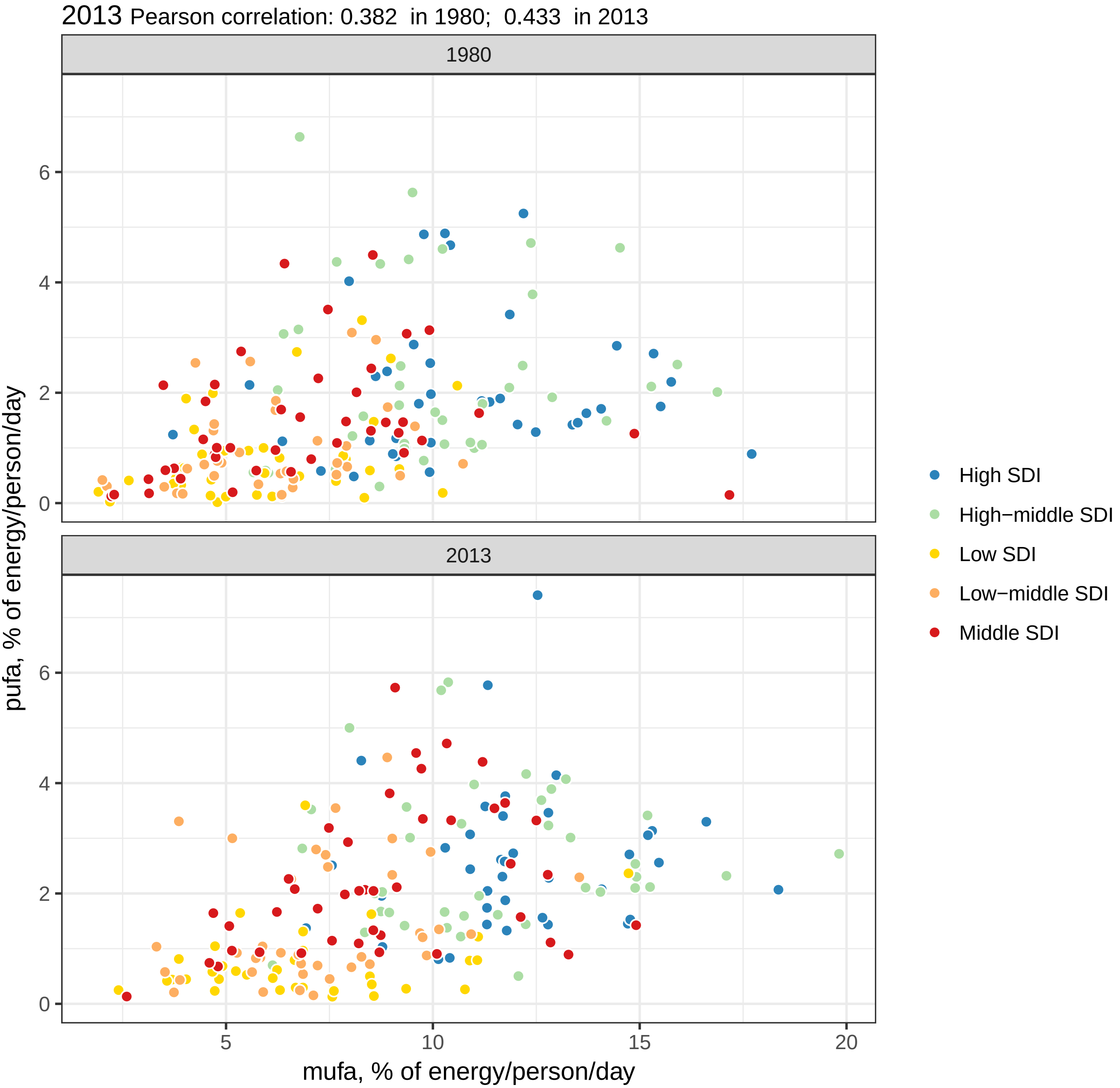
2013 Pearson correlation: 0.382 in 1980; 0.433 in 2013
1980
2013
pufa, % of energy/person/day
mufa, % of energy/person/day
0
2
4
6
5
10
15
20
High SDI
High−middle SDI
Low SDI
Low−middle SDI
Middle SDI

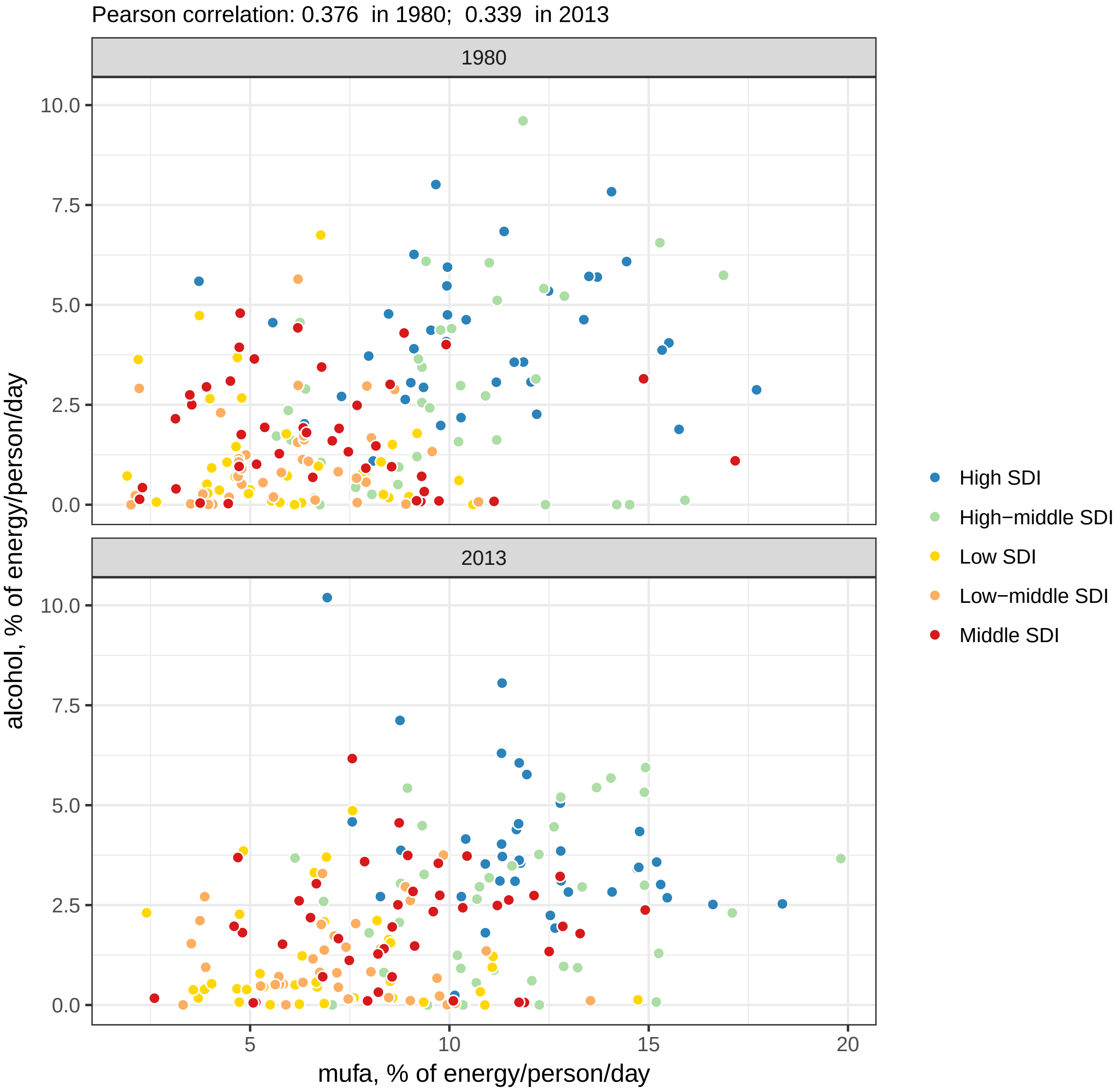

Pearson correlation: 0.376 in 1980; 0.339 in 2013
1980
2013
10.0
7.5
5.0
2.5
0.0
alcohol, % of energy/person/day
5
10
15
20
mufa, % of energy/person/day
High SDI
High−middle SDI
Low SDI
Low−middle SDI
Middle SDI

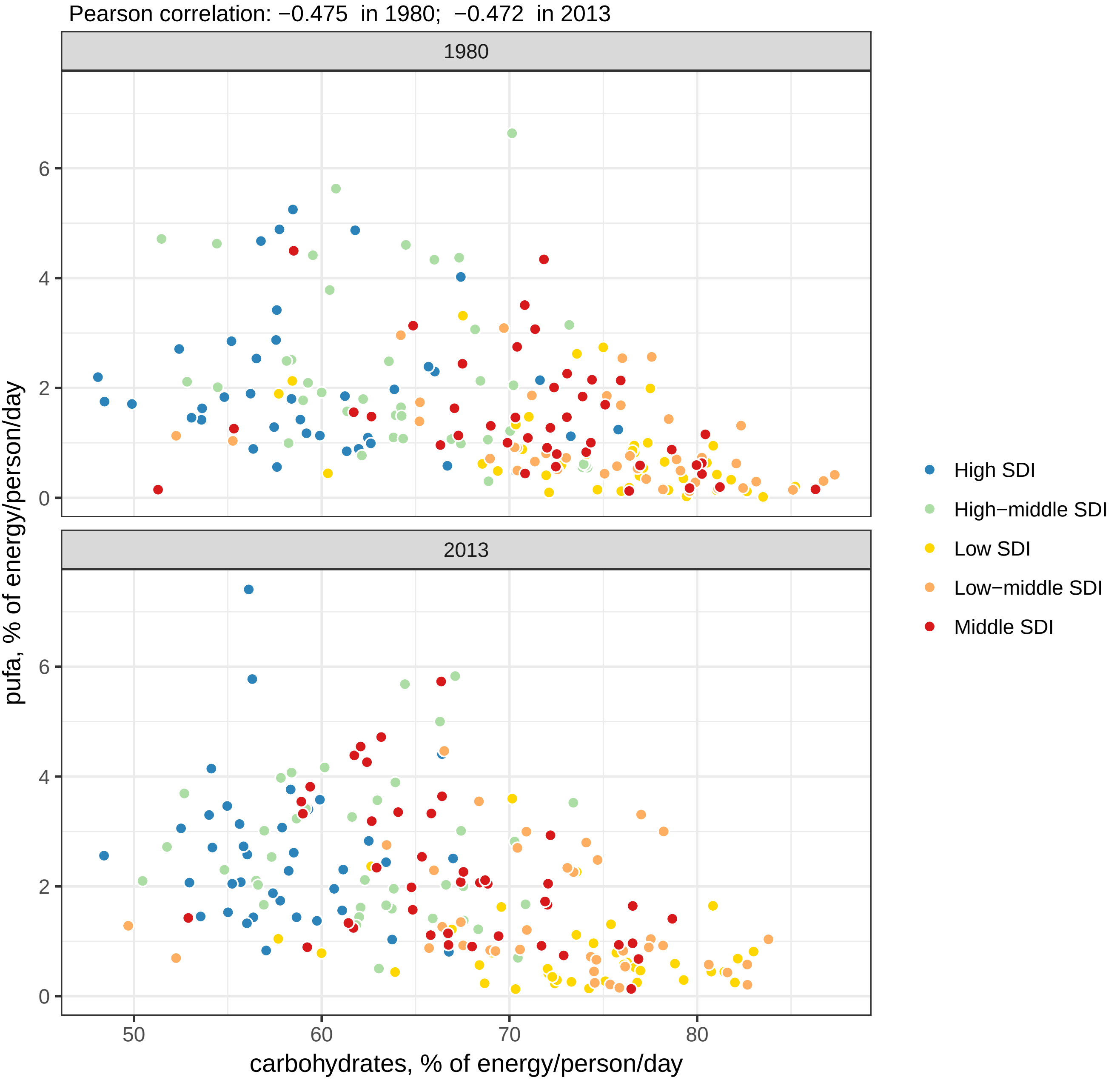

Pearson correlation: −0.475 in 1980; −0.472 in 2013
1980
2013
pufa, % of energy/person/day
carbohydrates, % of energy/person/day
0
2
4
6
50
60
70
80
High SDI
High−middle SDI
Low SDI
Low−middle SDI
Middle SDI

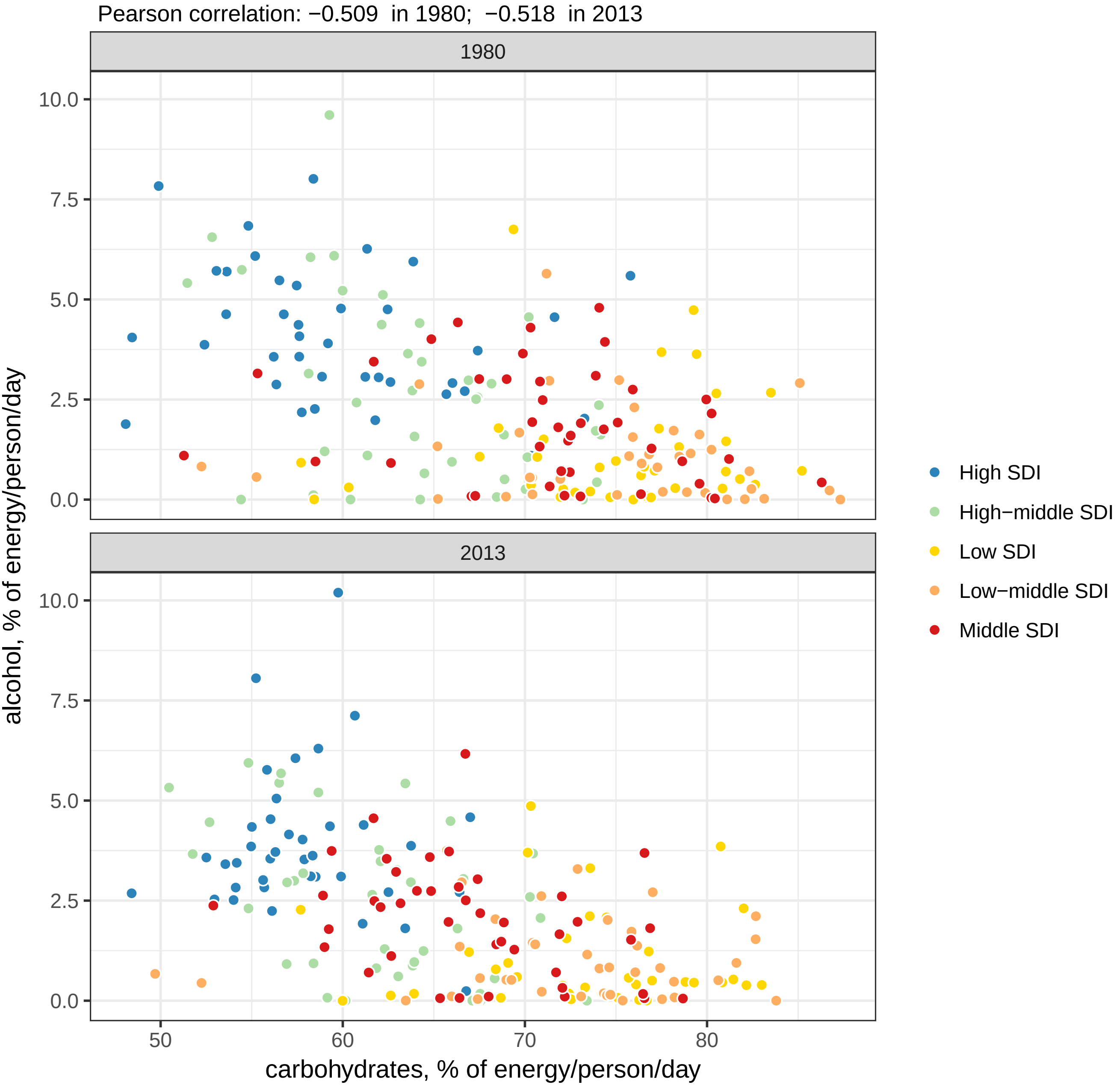

Pearson correlation: −0.509 in 1980; −0.518 in 2013
1980
2013
alcohol, % of energy/person/day
carbohydrates, % of energy/person/day
0.0
2.5
5.0
7.5
10.0
50
60
70
80
High SDI
High−middle SDI
Low SDI
Low−middle SDI
Middle SDI

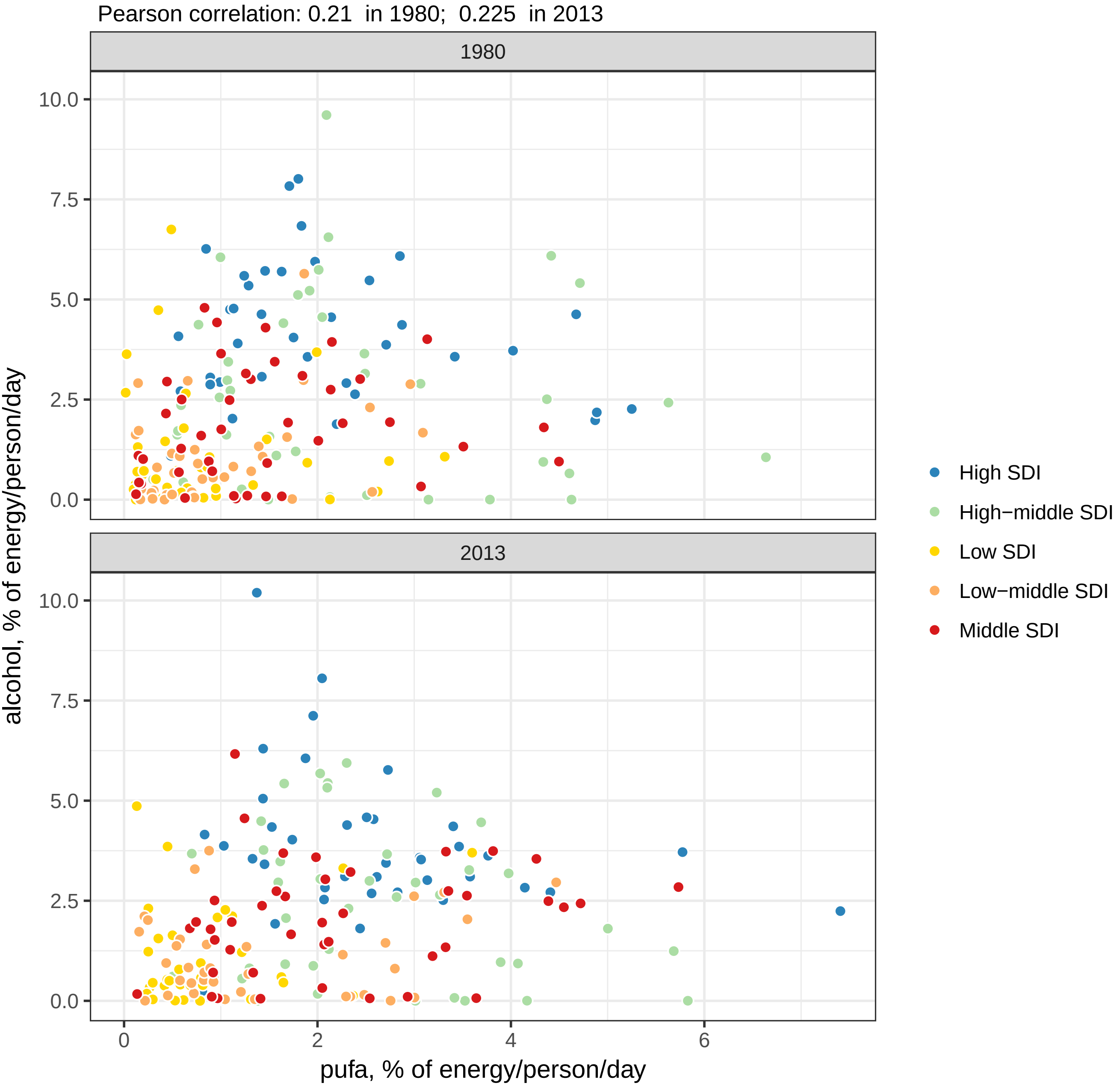
Pearson correlation: 0.21 in 1980; 0.225 in 2013
1980
2013
alcohol, % of energy/person/day
pufa, % of energy/person/day
0.0
2.5
5.0
7.5
10.0
0
2
4
6
High SDI
High−middle SDI
Low SDI
Low−middle SDI
Middle SDI

Supplemental Figure 2. Comparsion of percent of energy from macronutrients in Global Nutrient Database and The National Health and Nutrition Examination Survey (NHANES)

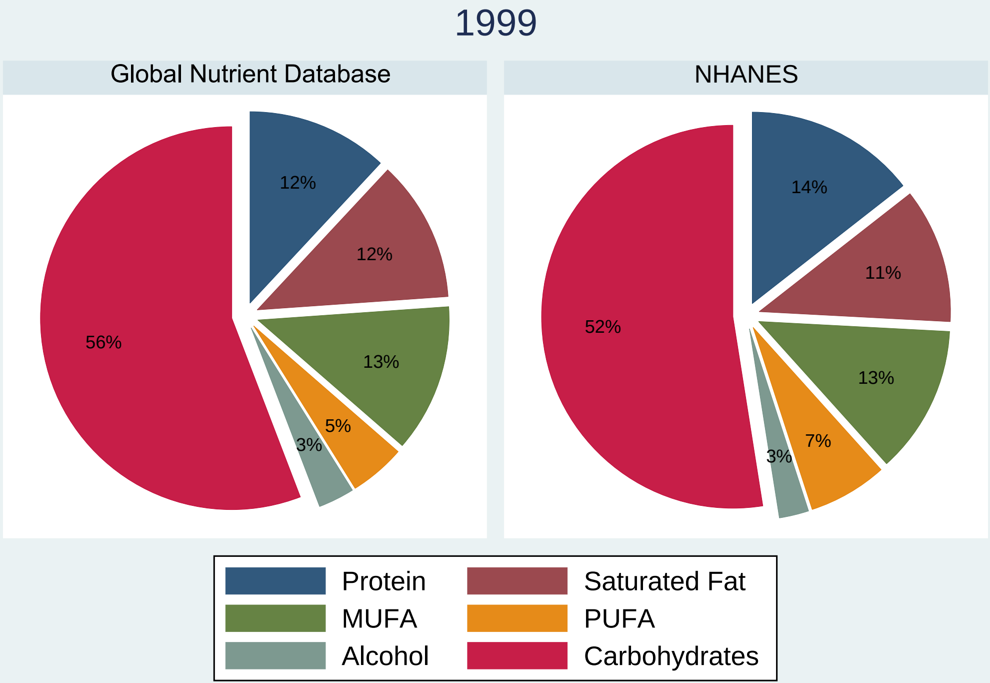

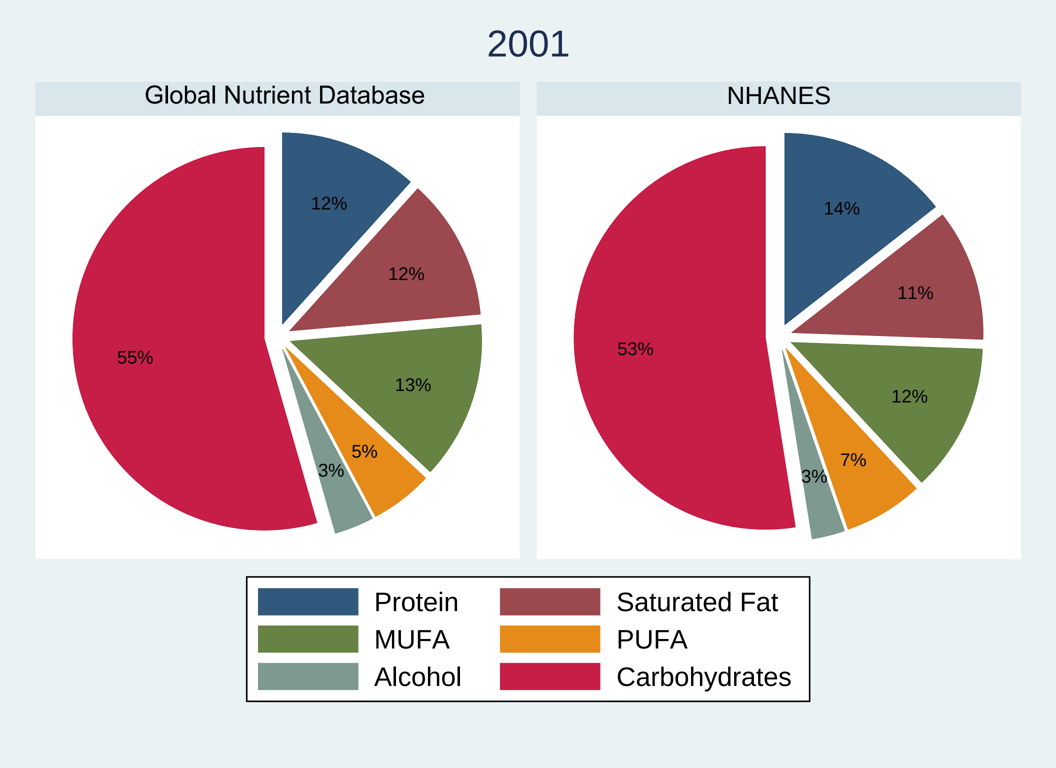
2001
Global Nutrient Database
NHANES
12%
12%
55%
13%
5%
3%
14%
11%
53%
12%
7%
3%
Protein
Saturated Fat
MUFA
PUFA
Alcohol
Carbohydrates

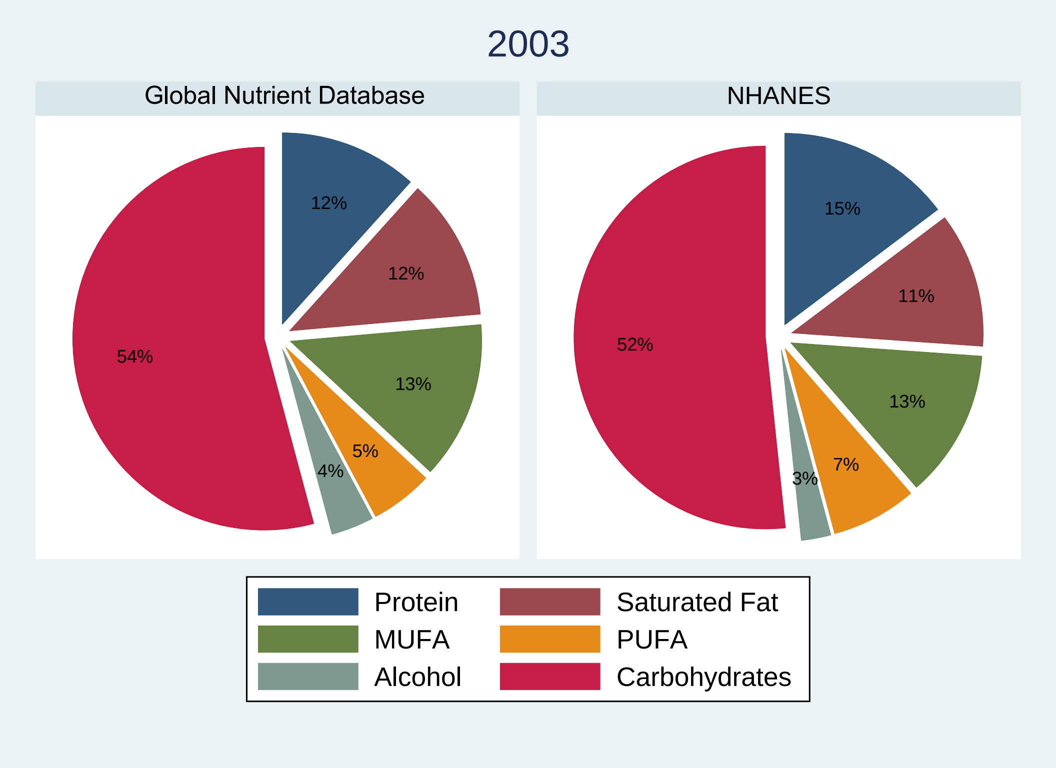
2003
Global Nutrient Database
12%
12%
13%
5%
4%
54%
NHANES
15%
11%
13%
7%
3%
52%
Protein
Saturated Fat
MUFA
PUFA
Alcohol
Carbohydrates

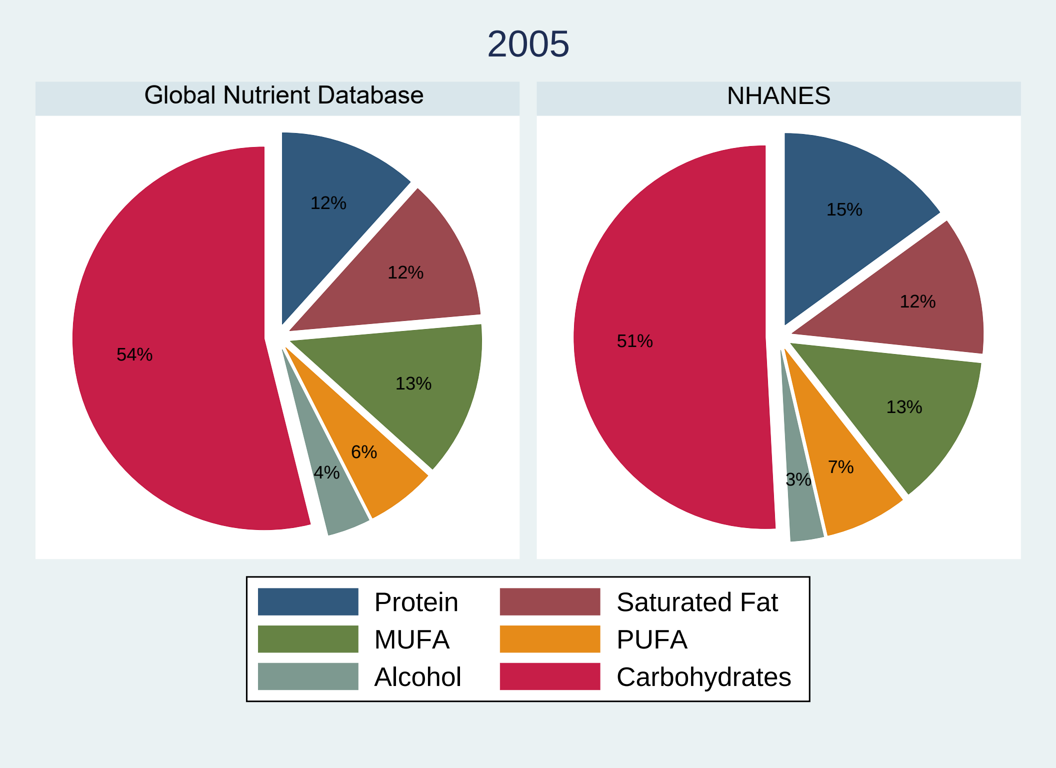
2005
Global Nutrient Database
12%
12%
13%
6%
4%
54%
NHANES
15%
12%
13%
7%
3%
51%
Protein
Saturated Fat
MUFA
PUFA
Alcohol
Carbohydrates

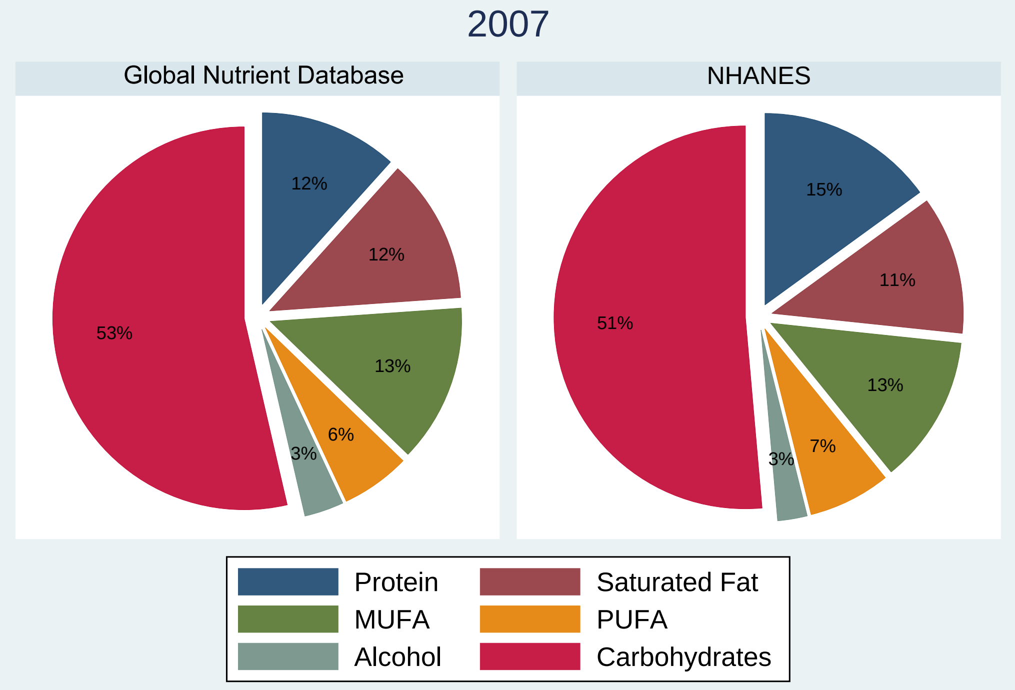
2007
Global Nutrient Database
NHANES
12%
12%
13%
6%
3%
53%
15%
11%
13%
7%
3%
51%
Protein
Saturated Fat
MUFA
PUFA
Alcohol
Carbohydrates

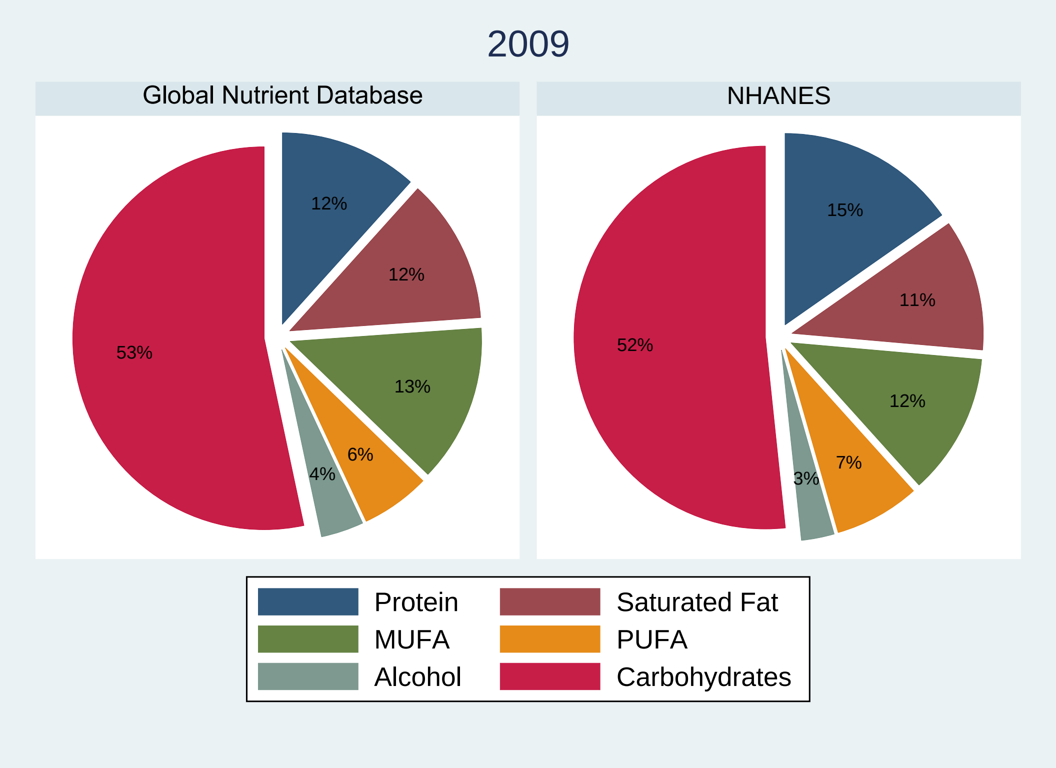
2009
Global Nutrient Database
12%
12%
13%
6%
4%
53%
NHANES
15%
11%
12%
7%
3%
52%
Protein
Saturated Fat
MUFA
PUFA
Alcohol
Carbohydrates

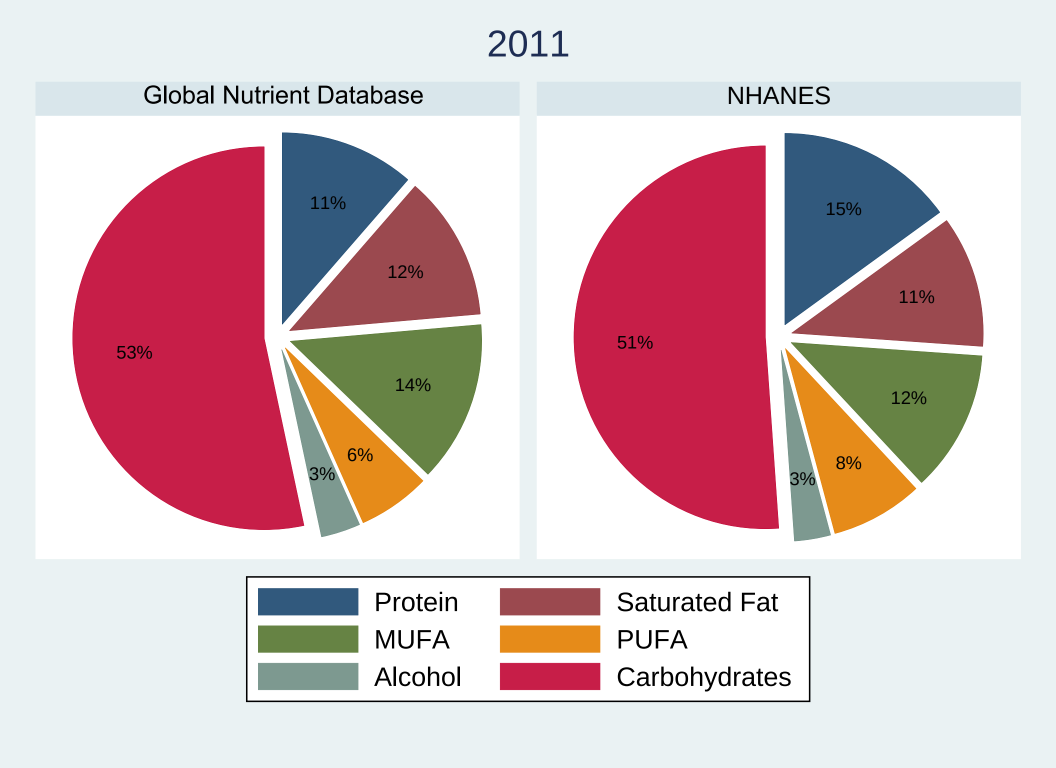
2011
Global Nutrient Database
NHANES
11%
12%
14%
6%
3%
53%
15%
11%
12%
8%
3%
51%
Protein
Saturated Fat
MUFA
PUFA
Alcohol
Carbohydrates

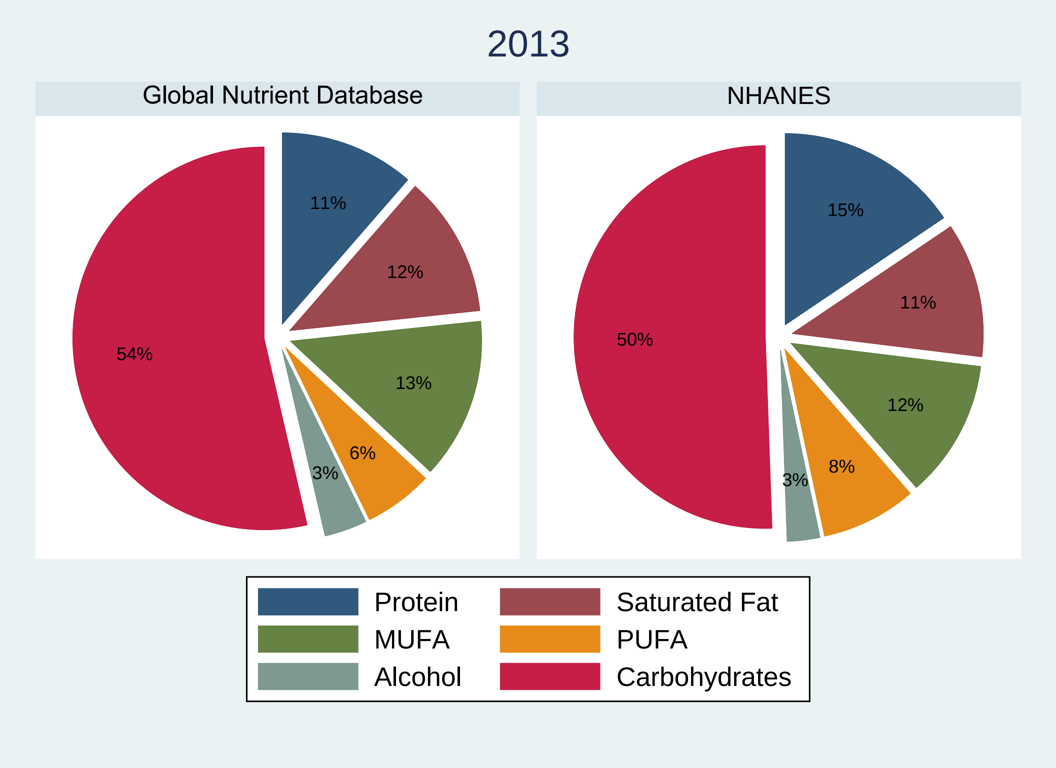
2013
Global Nutrient Database
11%
12%
13%
6%
3%
54%
NHANES
15%
11%
12%
8%
3%
50%
Protein
Saturated Fat
MUFA
PUFA
Alcohol
Carbohydrates
